# Association between anticholinergic burden and dementia in UK Biobank

**DOI:** 10.1002/trc2.12290

**Published:** 2022-04-12

**Authors:** Jure Mur, Tom C. Russ, Simon R. Cox, Riccardo E. Marioni, Graciela Muniz‐Terrera

**Affiliations:** ^1^ Lothian Birth Cohorts Group Department of Psychology University of Edinburgh Edinburgh UK; ^2^ Centre for Genomic and Experimental Medicine Institute of Genetics and Molecular Medicine University of Edinburgh Edinburgh UK; ^3^ Alzheimer Scotland Dementia Research Centre University of Edinburgh Edinburgh UK; ^4^ Edinburgh Dementia Prevention University of Edinburgh Edinburgh UK; ^5^ Division of Psychiatry Centre for Clinical Brain Science University of Edinburgh Edinburgh UK; ^6^ Centre for Genomic and Experimental Medicine Institute of Genetics and Cancer University of Edinburgh Edinburgh UK

**Keywords:** anticholinergic drugs, cohort study, dementia, prescriptions drugs, primary care

## Abstract

**Background:**

Previous studies on the relationship between anticholinergic drugs and dementia have reported heterogeneous results. This variability could be due to different anticholinergic scales and differential effects of distinct classes of drugs.

**Methods:**

Using Cox proportional hazards models, we computed the association between annual anticholinergic burden (AChB) and the risk of dementia in UK Biobank with linked general practitioner prescription records between the years 2000 and 2015 (n = 171,775).

**Results:**

AChB according to most anticholinergic scales (standardized odds ratio range: 1.027–1.125) and the slope of the AChB trajectory (hazard ratio = 1.094; 95% confidence interval: 1.068–1.119) were predictive of dementia. However, the association between AChB and dementia held only for some classes of drugs, especially antidepressants, antiepileptics, and antidiuretics.

**Discussion:**

The heterogeneity in previous findings may partially be due to different effects for different classes of drugs. Future studies should establish differences in more detail and further examine the practicality of a general measure of AChB relating to the risk of dementia.

## 1 INTRODUCTION

The number of people with dementia is predicted to increase in the UK by 50% from the year 2016 to 2040 and worldwide from 50 million today to 152 million in 30 years.^1^ Considering the lack of treatment options, the specification of risk factors to reduce the incidence of the disease is crucial. It is estimated that ≈40% of risk factors for dementia are preventable^1^ and that the decreases in the incidence of dementia in some countries are partially attributable to reductions in some of these risk factors.^2^

Anticholinergic drugs block muscarinic acetylcholine receptors in the nervous system, which are important in the innervation of brain areas involved in cognitive function and in the pathophysiology of Alzheimer's disease (AD).^3^ Due to their mechanism of action, sustained use of these medicines might impair cognitive function later in life. Anticholinergic burden (AChB)—a measure of anticholinergic drug use—has indeed been linked to an increased risk of cognitive impairment and dementia in older people.^4^, ^5^ Recent studies have focused on the long‐term effects of anticholinergic drugs when taken before advanced age: both Coupland et al.^6^ and Richardson et al.^7^ studied the associations between anticholinergic use in middle‐aged patients from general practices in the UK and the risk of late‐life dementia. For certain anticholinergic drugs, these studies reported an increased rate of dementia after their use decades before the diagnosis. This suggests a potential for AChB as a marker for cognitive decline or as a causative risk factor. In other words, AChB could be indicative of comorbidities that themselves affect cognition or could—through the drugs’ mechanism of action—contribute to cognitive decline as an independent risk factor. However, the status of anticholinergic medication in dementia prevention is unclear, as several recent reviews on the topic report heterogeneous findings.^4^, ^8^, ^9^

The variability in previous findings can be partially explained by differences in study design, the characteristics of the samples, the covariates in the models, and the choice of anticholinergic scales that assign drugs their anticholinergic potency. There is no widely accepted procedure to score anticholinergic potency^10^ and anticholinergic scales were constructed in distinct regions and contexts, and validated in different samples. Additionally, likely due to their propensity to cross the blood‐brain barrier, drugs differ in their ability to cause effects in the central nervous system as opposed to the periphery. Because anticholinergic scales are constructed with different outcomes in mind, they will not all place the same focus on centrally acting compounds. The above differences between scales lead to poor agreement between them and to uncertainty when choosing a scale for medical practice or research.^11^, ^12^

Moreover, the associations between anticholinergic drugs and dementia may hold only for some classes of drugs. Recent studies exploring class‐based associations reported effects especially for antidepressants, urological drugs, and antipsychotics.^6^, ^7^ Thus, while general recommendations of (de‐)prescribing of anticholinergic drugs are often made,^5^, ^6^, ^13^ they might not always be appropriate. This is especially the case because drugs are prescribed to manage underlying conditions that themselves decrease the quality of life and in cases when drug alternatives that exhibit fewer side effects are unavailable.

**RESEARCH IN CONTEXT**

1. Systematic Review: The authors used recent systematic reviews and manual search on PubMed to explore the extant literature. While there is research on the topic, the results are heterogeneous and only two studies probe the association between anticholinergic drug use in middle life and dementia in older age.
2. Interpretation: In this cohort study of 2124 participants diagnosed with dementia and 169,651 controls from UK Biobank, anticholinergic burden was associated with dementia risk across most scales used. However, only some drug classes were associated with dementia, especially antidepressants, antiepileptics, and antidiuretics. Anticholinergic potency did not show a clear relationship with dementia risk.
3. Future Directions: The relationships between various anticholinergic drugs and dementia should be clarified, and broad recommendations spanning several drug classes re‐evaluated.

To elucidate the proposed association between AChB and dementia, well‐powered replication studies and detailed inspections of the effects of different anticholinergic scales and drug classes are necessary. In this paper, the aims were threefold: (1) compare different anticholinergic scales in their propensity to predict dementia, (2) study the association between AChB at baseline and the longitudinal evolution of AChB and dementia, and (3) compare AChB due to different classes of drugs and the risk of dementia.

## 2 METHODS

### 2.1 Hypotheses

We expected AChB to be positively associated with dementia across anticholinergic scales and the association to be stronger than the association between dementia and polypharmacy. Furthermore, we anticipated the increase of AChB over time to be positively associated with dementia. Finally, based on previous studies,^6^, ^7^ we hypothesized that AChB due to antidepressants, antihistamines, antiepileptics, urological, and antipsychotic drugs would show a positive association with dementia. The association between other classes of drugs and dementia and the analysis of latencies between AChB and dementia was not based on prior hypotheses.

### 2.2 Sample

UK Biobank is a prospective study of > 500,000 participants that were recruited across the UK from 2006 to 2010.^14^ For ≈230,000 of these participants, primary‐care electronic prescription entries are available until September 2017. The entries contain the drugs prescribed, dates of prescriptions, and Read codes (https://isd.digital.nhs.uk^15^) that act as dictionaries for medicines. Diagnoses were obtained from two sources: (1) primary care electronic prescription records and (2) inpatient records. The former are prescriptions written on the computer by the primary care provider, while the latter are prescriptions issued during hospital stays. Dementia diagnoses and diagnoses used as covariates (see below) were ascertained using both primary care (UK Biobank field 42040) and hospital (UK Biobank fields 41270 and 41271) records (Table S1 in supporting information). In cases of multiple entries for a disorder, we retained the earliest record.

### 2.3 Anticholinergic burden and drug class

Eleven anticholinergic scales^16^, ^17^, ^18^, ^19^, ^20^, ^21^, ^22^, ^23^, ^24^, ^25^, ^26^ were chosen as previously identified^27^ and two^28^, ^29^ were identified through a recent systematic review.^30^ All anticholinergic scales used in this study, including full names and potential reasons for exclusion from the analyses, are listed in Table S2 in supporting information. One scale^25^ was modified to include newer drugs as before;^31^ for two scales,^17^, ^19^ updated versions were used (Aging Brain Care;^32^ Carnahan, 2014, personal communication on October 21, 2019). For one scale,^21^ drugs classified by the authors as having “improbable anticholinergic action” were assigned an anticholinergic burden of 0.5 (between “no anticholinergic potency” and “weak anticholinergic potency”) as has been done before.^27^

Using the British National Formulary (https://bnf.nice.org.uk^33^), brand names of anticholinergic drugs in the sample were substituted with generic names. Combination prescriptions containing several anticholinergic compounds were separated into multiple entries, each containing a single anticholinergic compound.

Each prescription was assigned anticholinergic scores based on the ratings from anticholinergic scales. Prescriptions of drugs with ophthalmic, otic, nasal, or topical routes of administration were assigned an anticholinergic score of 0, as before.^23^, ^24^, ^25^, ^26^ In the analysis comparing anticholinergic scales, for each scale, AChB was estimated by four separate means. First, the total yearly number of anticholinergic drugs was determined (count‐based scale). Second, each drug was assigned the anticholinergic value as listed in the anticholinergic scale and the values were summed for each year (value‐based scale). Third, a standardized dosage was calculated for each prescription by dividing the prescribed dose by the defined daily dose (DDD, https://www.whocc.no^34^) and then multiplying it by the anticholinergic score (dosage‐adjusted scale). Fourth, the quantity of the prescribed drug (e.g., volume or number of tablets) was accounted for by multiplying the prescribed dose with the quantity, divided by the DDD, and then multiplied by the anticholinergic score (quantity‐adjusted scale). To compare anticholinergic scales, separate models were run for each scale, resulting in 52 models. Additionally, two separate models were run for which polypharmacy was the main predictor. For the dosage‐adjusted and quantity‐adjusted scales, years in which any anticholinergic prescription was missing information on dosage or quantity, respectively, were removed for that participant (751 observations for the dosage‐adjusted scale and 8008 observations for the quantity‐adjusted scale). For all other analyses (i.e., when anticholinergic classes were not compared to one another), the scale by Dúran et al.^21^ was used to calculate AChB, as it exhibited the strongest association with the risk of dementia (see below). Each drug was assigned to a class based on the Anatomical Therapeutic Chemical (ATC) classification system (https://www.whocc.no/atc_ddd_index/^34^; Table S3 in supporting information) and to a group of anticholinergic potency (groups 0, 0.5, 1, 2; a higher value indicates a greater presumed anticholinergic potency) according to the anticholinergic scale by Dúran et al.^21^

### 2.4 Covariates and statistical analysis

The predictor in most models was the cumulative AChB in year 0 (the sum of anticholinergic scores of prescriptions for a participant). Due to the low ascertainment of prescriptions in the early years of sampling,^27^ year 0 was for each participant defined as the first full year of having been included in the prescriptions’ register after the year 1999.

Because the rate of dementia increases with age, participants younger than 60 years at the time of diagnosis or at the end of the prescriptions sampling period (June 30, 2020)—whichever came first—were excluded from the analyses. Additionally, participants who before year 0 or within a cut‐off period after year 0, had been diagnosed with dementia or prescribed a cholinesterase inhibitor (donepezil, galantamine, or rivastigmine) or memantine were excluded from the analyses. For all analyses in the main text, the cut‐off period above was 1 year. Based on comments by the reviewers, we varied this cut‐off and repeated the analysis on the association between AChB according to the scale by Dúran et al.^21^ and dementia for every possible value of this cut‐off (1 year to 20 years; Figure S4 in supporting information). People diagnosed with certain disorders are more likely to develop dementia. For this reason, we also excluded participants diagnosed at any point with Parkinson's disease, Huntington disease, Creutzfeldt‐Jacob disease, or multiple sclerosis from our analyses. Finally, the prescribing period after the year 2015 was incomplete^27^ and was removed. The data cleaning process is described in Figure S1 in supporting information.

Models were adjusted for age at year 0, sex (reference: female), data provider (region‐specific providers of prescriptions: The Phoenix Partnership [TPP] England, Vision England [reference], Vision/EMIS Health Scotland, Vision/EMIS Health Wales), education (binary; reference: no graduate degree), socioeconomic deprivation based on census data (scale range: –12 to 12; range in sample: –6.3 to 7.4; bigger number indicates greater deprivation),^35^ body mass index (BMI in kg/m^2^, categorized: < 18.5, 18.5–25 [reference], 25–30, 30–35, 35–40, > 40), self‐reported smoking status (smoker, non‐smoker [reference], former smoker), self‐reported alcohol consumption frequency (daily or almost daily [reference], three or four times a week, once or twice a week, one to three times a month, only on special occasions, never), self‐reported physical activity (mild [reference], moderate, strenuous),^36^ number of comorbidities (number of all unique diagnosis codes) by year 0, depression by year 0 (reference: no depression), stroke by year 0 (reference: no stroke), diabetes by year 0 (reference: no diabetes), hypercholesterolemia by year 0 (reference: no hypercholesterolemia), hypertension by year 0 (reference: no hypertension), apolipoprotein E (*APOE*) carrier status (reference: ε2), and polypharmacy. The latter was determined separately for each anticholinergic scale by subtracting the yearly number of anticholinergic drugs according to that scale from the total yearly drug count. *APOE* genotype was determined based on the nucleotides at single nucleotide polymorphism positions rs239358 and rs7412; *APOE* carrier status was denoted as ε3 for participants with the ε3/ε3, ε1/ε3, or ε2/ε4 haplotype, ε2 for participants with the ε2/ε2 or ε2/ε3 haplotype, and ε4 for participants with the ε3/ε4 or ε4/ε4 haplotype.

For the association between AChB and dementia, Cox proportional hazards models were used, and effects are expressed as hazard ratios (HRs) with accompanying 95% confidence intervals (CIs). For studying time‐to‐event latencies, logistic regression was used, and effects are expressed in odds ratios (ORs). The association between the longitudinal evolution of AChB and dementia accounted for the competing risk of death and was assessed with the joint model for longitudinal and time‐to‐event data using the R library JM.^37^ For all other analyses using only a single anticholinergic scale, the value‐based scale by Durán et al.^21^ was used, as it exhibited the strongest association with dementia. Models for which AChB was the main predictor were additionally controlled for polypharmacy. The two models for which polypharmacy was the main predictor differed from each other in the included covariates: (1) one was controlled for all covariates described above except for polypharmacy, and (2) the other (termed “polypharmacy plus”) was additionally controlled for the total number of anticholinergic drugs (according to any anticholinergic scale).

Numerical values three or more standard deviations beyond the mean were defined as outliers and removed from the analytical sample prior to analysis. Due to zero inflation for AChB, the number of prescriptions, and the number of comorbidities, null values were removed before calculating means and standard deviations for outlier removal for these variables. Cases with missing values were removed prior to analysis and constituted up to 16.9% of the sample, depending on the model. When exploring the AChB attributable to different drug classes, only drug classes were included that were in year 0 prescribed to at least 10 participants that later developed dementia. The proportional hazards assumption was satisfied, but the assumption of linearity between the predictor and the log hazard was sometimes violated (Figure S2 in supporting information). In models in which that was the case, the covariates were transformed, and the type of transformation is indicated in the results. When a distinct model was run for each predictor, the Bonferroni correction was used. When all predictors were included in a single model, no adjustment for multiple comparisons was done. Numerical variables were scaled to have a mean of 0 and a standard deviation of 1. Results are reported as standardized effect sizes. All analyses were performed in R version 4.1.0 and Python 3.7.10. The code is available at https://github.com/JuM24/UKB‐ACB‐dementia.

## 3 RESULTS

### 3.1 Characteristics of the sample

After data cleaning, the final sample consisted of 171,775 participants. Among the participants, 2124 (1.2%) were diagnosed with dementia (Table S4 in supporting information), with diagnoses dating between July 2002 and June 2020. The median age of participants at year 0 was 55 years (Q1 = 49 years, Q3 = 59 years) and the median age of diagnosis with dementia was 72.6 years (interquartile range [IQR] = 7.2). The average follow‐up—defined as the median number of years between year 0 and the year of censoring—was 20 years for participants without dementia (Q1 = 14 years, Q3 = 20 years) and 14 years for participants diagnosed with dementia (Q1 = 11 years, Q3 = 17 years). The characteristics of variables for year 0 are presented in Table 1 and Table S5 in supporting information. Depending on the scale used, anticholinergic drugs constituted between 2.5% and 21.8% of all prescriptions between the years 2000 and 2015, with 0.24 to 2.12 anticholinergic prescriptions per person in year 0 (Table S6 in supporting information). The characteristics of anticholinergic prescribing in UK Biobank have been described in greater detail elsewhere.^27^

**TABLE 1:** : Descriptive statistics of variables used in the models

**Variable**	**Level**	**Median (IQR) or n (%)**
Age		55 (10)
Sex	Female	94,310 (54.9)
Education	No graduate degree	118,191 (69.7)
Deprivation		–2.3 (3.8)
Alcohol consumption	Daily or almost daily	35,989 (21.0)
	Three or four times a week	39,747 (23.2)
	Once or twice a week	43,815 (25.6)
	Once to three times a month	18,149 (10.6)
	Only special occasions	19,673 (11.5)
	Never	14,024 (8.2)
Smoking	Current smoker	16,412 (9.6)
	Previous smoker	63,372 (37.1)
	Non‐smoker	91,091 (53.3)
Physical activity	Strenuous	13,577 (8.5)
	Moderate	103,121 (64.7)
	Light	42,777 (26.8)
BMI	<18.5	768 (0.45)
	18.5–25	3372 (2.0)
	25–30	51,649 (30.2)
	30–35	74,192 (43.4)
	35–40	31,807 (18.6)
	>40	9070 (5.3)
Data provider	England (Vision)	14,036 (8.2)
	Scotland	18,758 (10.9)
	England (TPP)	123,133 (71.7)
	Wales	15,848 (9.2)
Dementia diagnosis		2124 (1.2)
Prior depression		13,136 (7.6)
Prior stroke		1598 (0.9)
Prior diabetes		4034 (2.3)
Prior hypercholesterolemia		4901 (2.9)
Prior hypertension		16,152 (9.4)
Number of prior comorbidities		18 (40)
Total number of prescriptions^*^		3 (12)
*APOE* carrier	ε2	21,626 (12.9)
	ε3	102,740 (61.3)
	ε4	43,199 (25.8)

* The total number of prescriptions was used along the number of anticholinergic drugs to calculate the scale‐specific non‐anticholinergic drug count.

Abbreviations: *APOE*, apolipoprotein E; BMI, body mass index; IQR, interquartile range; TPP, The Phoenix Partnership.

### 3.2 Anticholinergic scales comparison

**FIGURE 1:**
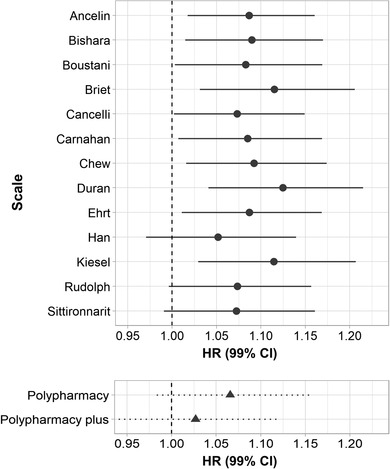
Hazard ratios (HRs) for the association between anticholinergic burden (top panel) or drug count (bottom panel) and dementia. The names on the y‐axis of the top panel refer to the first names of the authors of the original anticholinergic scales; “polypharmacy plus” was additionally controlled for the total number of anticholinergic drugs. CI, confidence interval

Most anticholinergic scales showed positive associations with dementia and with greater effect size estimates than for general polypharmacy (Figure 1). HRs for standardized AChB ranged from 1.027 to 1.125 (count‐based: median = 1.087, IQR = 0.044; value‐based: median = 1.087, IQR = 0.019; dosage‐adjusted: median = 1.078, IQR = 0.009; quantity‐adjusted: median = 1.065, IQR = 0.032; Tables S7 and S8 in supporting information). The overlap in CIs was substantial both between scales and within scales; similar results were observed for models with log‐ and rank‐inverse normally transformed predictors (Figure S3 in supporting information). The value‐based scale by Durán et al.^21^ exhibited the strongest association with dementia (Tables S7 and S9 in supporting information) and was used in all subsequent analyses. The effect of AChB on dementia was relatively invariant among the models with different exclusion cut‐offs for the period of dementia diagnosis (Figure S4 in supporting information). When death was modeled as a competing outcome, a one standard deviation increase in AChB was associated with a 12.0% (95% CI: 7.1%–17.2%) increase in the incidence of dementia, and a 6.0% (95% CI: 3.5%–8.5%) increase in the incidence of all‐cause mortality.

### 3.3 Time‐to‐event latency

We compared the risk of dementia occurring within 12 years, between 12 and 14 years, between 14 and 16 years, 16 and 18 years, or more than 18 years (effectively 18–20.5) after year 0. ORs did not differ between most of the different latencies, nor was a pattern discernible in the relationship between latency and effect size (Table 2).

**TABLE 2:** : ORs for the risk of dementia within different time periods since the measurement of anticholinergic burden

**Latency** (years since 2000)	**OR**	**95% CI**	**N cases**
0–12	1.20	1.05–1.34	250
12–14	1.06	0.90–1.22	257
14–16	1.11	1.00–1.22	446
16–18	1.21	1.10–1.33	375
18–20.5	1.07	0.98–1.15	813

Abbreviations: CI, confidence interval; OR, odds ratio.

### 3.4 Change in AChB and dementia

The estimate for the association between the individual longitudinal evolution of AChB and dementia was positive (HR = 1.094; 95% CI: 1.068–1.119). When the rate of dementia was modeled as a function of the individual longitudinal evolution of AChB in a competing risk model (competing risks: dementia, death), the effect was also positive (death: HR = 1.066, 95% CI = 1.042–1.089; dementia: HR = 1.056, 95% CI = 1.008–1.11).

### 3.5 Drug classes and categories of AChB

**FIGURE 2:**
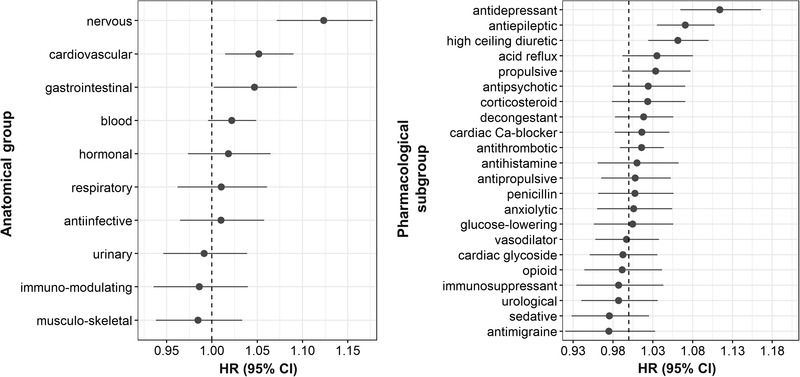
Hazard ratios (HRs) for the association between anticholinergic burden (rank‐based inverse normal transformation) attributable to different classes of drugs and dementia. Left and right panels reflect the same data, but at different levels of granularity, with left panel representing the topmost level, and right panel the third level from the top according to the World Health Organization classification. CI, confidence interval

**FIGURE 3:**
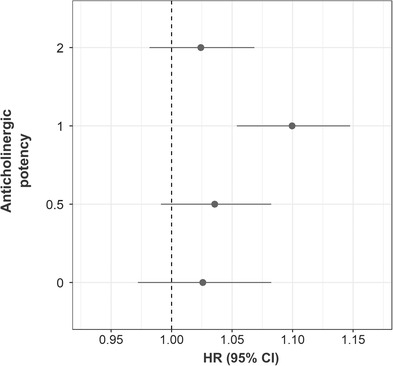
Hazard ratios (HRs) for the association between the numbers of anticholinergic drugs (rank‐based inverse normal transformation) of different levels of potency and dementia. CI, confidence interval

Several drug classes exhibited a positive association between AChB and dementia, including drugs for treating the nervous‐, gastrointestinal‐, and cardiovascular systems (Figure 2; Tables S10, S11 in supporting information). The effect was strongest for antiepileptic drugs, antidepressants, and diuretics (furosemide). While many drugs exhibited a positive tendency for an association between AChB and dementia, the effect sizes were small, and the CIs mostly overlapped with HR = 1. When the individual yearly drug counts for each group of anticholinergic potency were used to predict dementia (Figure 3; Table S12 in supporting information), only the number of drugs with an anticholinergic potency of 1 was predictive of dementia.

## 4 DISCUSSION

### 4.1 Interpretation of the findings

In this study, we used electronic prescription data from 171,775 participants in UK Biobank to study the relationship between AChB and dementia risk. In line with our hypotheses, AChB was associated with dementia across most anticholinergic scales and the best effect estimate for most scales tended to be greater than that for polypharmacy. The data also supported our hypothesis that the trajectory of AChB over time was predictive of dementia, even after accounting for the competing risk of death. The hypotheses regarding class‐specific effects were mostly upheld, with AChB due to antidepressants, antiepileptics, and antihistamines positively associated with dementia risk. However, the effects for antipsychotics and for urological drugs were not significant. We also found associations between additional classes of drugs and risk of dementia, especially high‐ceiling diuretics (furosemide). Finally, the strength of the association between AChB and dementia remained unchanged, regardless of the latency between time of measurement and time of diagnosis.

Our results support an association between AChB and dementia across anticholinergic scales, a finding observed previously using self‐reported medicine use in UK Biobank.^36^ This relationship persisted after controlling for several covariates. Across most anticholinergic scales, AChB was a stronger predictor than the total number of prescribed drugs, suggesting that anticholinergic medicines may represent a risk factor distinct from polypharmacy. When applying the anticholinergic scale^21^ that exhibited the strongest association with dementia, AChB also predicted all‐cause mortality. Furthermore, not only cumulative AChB measured over 1 year, but the intra‐individual longitudinal trajectory in AChB over the course of 15 years was associated with the risk of dementia. In other words, steeper slopes in the increase of AChB over time were associated with an increased risk of dementia.

However, despite the association between AChB and dementia, several caveats need consideration. First, in contrast to previous findings^6^, ^7^, ^38^ suggesting a dose–response relationship, including dosage and quantity in the computation of AChB did not increase model precision or the strength of the association between AChB and dementia. The same was true for the inclusion of anticholinergic scores: simply counting anticholinergic drugs (as opposed to assigning a potency value or weighing by dosage) was equally predictive of dementia. Second, the association between AChB and dementia was limited to AChB attributable to certain classes of drugs. This is consistent with previous findings^6^, ^7^ that reported that AChB attributable to antidepressants, antihistamines, and antiepileptic drugs was associated with dementia; this consistency was not found for antipsychotics and urological drugs. Third, findings here and elsewhere^7^ indicate that a higher anticholinergic potency of a drug does not always correspond to a higher risk of dementia.

The consistency in effect sizes for the association between AChB and dementia for different time‐to‐event latencies has been observed before^6^, ^7^ and suggests that the value of AChB as a potential marker of later cognitive decline does not vary with time. This could indicate the longitudinal consistency in differences in AChB between individuals. While some authors^7^ understand this finding as strengthening the case for causality, it could also—along with the primary finding of an association between AChB and dementia—be explained by confounding by indication: dementia could be caused by the indication for which anticholinergic drugs were prescribed. Indeed, the drugs classes linked to dementia in our study and others^6^, ^7^ are used to treat cardiovascular problems, epilepsy, depression, and schizophrenia, which themselves correlate with neuroanatomical changes or may act as risk factors for dementia.^1^, ^39^, ^40^, ^41^, ^42^, ^43^ However, the lack of differences in effect size for various latencies does not preclude causality between AChB and dementia. As opposed to increasing the rate of cognitive decline (i.e., the slope of longitudinal cognitive function), the results could be explained by AChB producing a fixed degree of cognitive impairment (i.e., change the intercept of longitudinal cognitive function).

### 4.2 Strengths and weaknesses

The main strengths of our study are the size of the sample, the depth of available data, and the high accuracy of UK Biobank for ascertainment of dementia.^44^ Furthermore, our analyses examined AChB from multiple perspectives, including comparing different scales and drug classes. However, we acknowledge several limitations. The participants in UK Biobank are on average healthier and live in less deprived areas than the UK population.^45^ Additionally, linked data do not include information on over‐the‐counter drugs and dietary supplements. Thus, AChB in the UK is likely higher than estimated in our study. Also, due to the low average age of the participants, UK Biobank has relatively few cases of dementia. Next, our analytical approach exhibits weaknesses. First, the dosages and quantities of medicines used in the calculation of the dosage‐ and quantity‐adjusted scales required substantial manual cleaning and may not have been completely accurate. Second, the assumption of linearity between the predictor and the log hazard was sometimes not satisfied and transformations of the data were required to reliably run the models. Third, comparing the effects of different potencies of anticholinergic drugs, prescriptions with the highest potency were much less common than other groups of drugs. This could have affected the accuracy of our estimate.

## 5 CONCLUSIONS AND FUTURE DIRECTIONS

Inconsistencies in the literature, uncertainty of dose–response‐ or potency–response relationships, a strong drug‐class dependency, and the difficulty of excluding confounding by indication, have led some^46^ to suggest that a different common denominator—other than anticholinergic effect—is responsible for the observed association between anticholinergic drugs and dementia. If correct, the first goal should be the elucidation of the proposed association. Instead of studying the relationship of a general measure of AChB and cognitive decline, researchers could specify and describe the role of distinct classes of anticholinergic medicines—or even individual drugs.

Considering the role of the cholinergic system in the development of AD,^3^ a biological underpinning for the effect of anticholinergic drugs in dementia is intuitive. However, further evidence is needed to determine the brain regions associated with the action of these drugs and the biological pathways likely involved in their proposed effects.

Finally, while previous studies assessed and/or compared anticholinergic scales,^12^, ^30^, ^47^, ^48^, ^49^, ^50^ questions about their relevance and potential utility remain unanswered. Scales are most often constructed based on expert opinions rooted in past practice and propound established views that might be dated. The contents of anticholinergic scales may certainly reflect a facet of inappropriate prescribing that could help in medical decision‐making. However, their heterogeneity and lack of a clear potency–outcome relationship point to an urgent need for reappraisal.

## CONFLICTS OF INTEREST

REM has received consulting fees from the Epigenetic Clock Development Foundation and speaker fees from Illumina. TCR has received fees for medicolegal work from private solicitors. SRC has received speaker fees from the Society of Biological Psychiatry. GMT has received consulting fees for grants funded by the NIH. JM has nothing to disclose.

## Supporting information

Supporting Information

Supporting Information

Supporting Information

## References

[1] Livingston G , Huntley J , Sommerlad A , et al. Dementia prevention, intervention, and care: 2020 report of the Lancet Commission. The Lancet. 2020;396(10248):413‐446. 10.1016/S0140-6736(20)30367-6 PMC7392084 32738937

[2] Wu YT , Beiser AS , Breteler MMB , et al. The changing prevalence and incidence of dementia over time ‐ current evidence. Nat Rev Neurol. 2017;13(6):327‐339. 10.1038/nrneurol.2017.63 28497805

[3] Hampel H , Mesulam MM , Cuello AC , et al. The cholinergic system in the pathophysiology and treatment of Alzheimer's disease. Brain. 2018;141(7):1917‐1933. 10.1093/brain/awy132 29850777 PMC6022632

[4] Taylor‐Rowan M , Edwards S , Noel‐Storr AH , et al. Anticholinergic burden (prognostic factor) for prediction of dementia or cognitive decline in older adults with no known cognitive syndrome. Cochrane Database Syst Rev. 2021;5:CD013540. 10.1002/14651858.CD013540.pub2 34097766 PMC8169439

[5] Zheng YB , Shi L , Zhu XM , et al. Anticholinergic drugs and the risk of dementia: A systematic review and meta‐analysis. Neurosci Biobehav Rev. 2021;127:296‐306. 10.1016/j.neubiorev.2021.04.031 33933505

[6] Coupland CAC , Hill T , Dening T , Morriss R , Moore M , Hippisley‐Cox J . Anticholinergic drug exposure and the risk of dementia: a nested case‐control study. JAMA Intern Med. 2019;179(8):1084‐1093. 10.1001/jamainternmed.2019.0677 31233095 PMC6593623

[7] Richardson K , Fox C , Maidment I , et al. Anticholinergic drugs and risk of dementia: case‐control study. BMJ. 2018;361:k1315. 10.1136/bmj.k1315 29695481 PMC5915701

[8] Andre L , Gallini A , Montastruc F , et al. Association between anticholinergic (atropinic) drug exposure and cognitive function in longitudinal studies among individuals over 50 years old: a systematic review. Eur J Clin Pharmacol. 2019;75(12):1631‐1644. 10.1007/s00228-019-02744-8 31468067

[9] Pieper NT , Grossi CM , Chan WY , et al. Anticholinergic drugs and incident dementia, mild cognitive impairment and cognitive decline: a meta‐analysis. Age Ageing. 2020;49(6):939‐947. 10.1093/ageing/afaa090 32603415 PMC7583519

[10] Rudd KM , Raehl CL , Bond CA , Abbruscato TJ , Stenhouse AC . Methods for assessing drug‐related anticholinergic activity. Pharmacotherapy. 2005;25(11):1592‐1601. 10.1592/phco.2005.25.11.1592 16232021

[11] Naples JG , Marcum ZA , Perera S , et al. Concordance between anticholinergic burden scales. J Am Geriatr Soc. 2015;63(10):2120‐4. 10.1111/jgs.13647 26480974 PMC4617193

[12] Welsh TJ , van der Wardt V , Ojo G , Gordon AL , Gladman JRF . Anticholinergic drug burden tools/scales and adverse outcomes in different clinical settings: a systematic review of reviews. Drugs Aging. 2018;35(6):523‐538. 10.1007/s40266-018-0549-z 29736815

[13] Dmochowski RR , Thai S , Iglay K , et al. Increased risk of incident dementia following use of anticholinergic agents: a systematic literature review and meta‐analysis. Neurourol Urodyn. 2021;40(1):28‐37. 10.1002/nau.24536 33098213 PMC7821204

[14] Sudlow C , Gallacher J , Allen N , et al. UK biobank: an open access resource for identifying the causes of a wide range of complex diseases of middle and old age. PLoS Med. 2015;12(3):e1001779. 10.1371/journal.pmed.1001779 25826379 PMC4380465

[15] NHS Digital Trud . Accessed February 9th 2022, 2022. https://isd.digital.nhs.uk

[16] Ancelin ML , Artero S , Portet F , Dupuy AM , Touchon J , Ritchie K . Non‐degenerative mild cognitive impairment in elderly people and use of anticholinergic drugs: longitudinal cohort study. BMJ. 2006;332(7539):455‐9. 10.1136/bmj.38740.439664.DE 16452102 PMC1382539

[17] Boustani M , Campbell N , Munger S , Maidment I , Fox C . Impact of anticholinergics on the aging brain: a review and practical application. Aging Health. 2008;4(3):311‐320. 10.2217/1745509X.4.3.311

[18] Cancelli I , Valentinis L , Merlino G , Valente M , Gigli GL . Drugs with anticholinergic properties as a risk factor for psychosis in patients affected by Alzheimer's disease. Clin Pharmacol Therapeut. 2008;84(1):63‐68. 10.1038/sj.clpt.6100435 17987049

[19] Carnahan RM , Lund BC , Perry PJ , Pollock BG , Culp KR . The Anticholinergic Drug Scale as a measure of drug‐related anticholinergic burden: associations with serum anticholinergic activity. J Clin Pharmacol. 2006;46(12):1481‐6. 10.1177/0091270006292126 17101747

[20] Chew ML , Mulsant BH , Pollock BG , et al. Anticholinergic activity of 107 medications commonly used by older adults. J Am Geriatr Soc. 2008;56(7):1333‐41. 10.1111/j.1532-5415.2008.01737.x 18510583

[21] Durán CE , Azermai M , Vander Stichele RH . Systematic review of anticholinergic risk scales in older adults. Eur J Clin Pharmacol. 2013;69(7):1485‐96. 10.1007/s00228-013-1499-3 23529548

[22] Ehrt U , Broich K , Larsen JP , Ballard C , Aarsland D . Use of drugs with anticholinergic effect and impact on cognition in Parkinson's disease: a cohort study. J Neurol Neurosurg Psychiatry. 2010;81(2):160‐5. 10.1136/jnnp.2009.186239 19770163

[23] Han L , McCusker J , Cole M , Abrahamowicz M , Primeau F , Élie M . Use of medications with anticholinergic effect predicts clinical severity of delirium symptoms in older medical inpatients. Archi Intern Med. 2001;161(8):1099‐1105. 10.1001/archinte.161.8.1099 11322844

[24] Kiesel EK , Hopf YM , Drey M . An anticholinergic burden score for German prescribers: score development. BMC Geriatr. 2018;18(1):239. 10.1186/s12877-018-0929-6 30305048 PMC6180424

[25] Rudolph JL , Salow MJ , Angelini MC , McGlinchey RE . The anticholinergic risk scale and anticholinergic adverse effects in older persons. Arch Intern Med. 2008;168(5):508‐513. 10.1001/archinternmed.2007.106 18332297

[26] Sittironnarit G , Ames D , Bush AI , et al. Effects of anticholinergic drugs on cognitive function in older Australians: results from the AIBL study. Dement Geriatr Cogn Disord. 2011;31(3):173‐8. 10.1159/000325171 21389718

[27] Mur J , Cox SR , Marioni RE , Muniz‐Terrera G , Russ TC . Increase in anticholinergic burden from 1990 to 2015: age‐period‐cohort analysis in UK biobank. Br J Clin Pharmacol. 2021; 10.1111/bcp.15045 34409635

[28] Bishara D , Harwood D , Sauer J , Taylor DM . Anticholinergic effect on cognition (AEC) of drugs commonly used in older people. Int J Geriatr Psychiatry. 2017;32(6):650‐656. 10.1002/gps.4507 27280553

[29] Briet J , Javelot H , Heitzman E , et al. The anticholinergic impregnation scale: towards the elaboration of a scale adapted to prescriptions in French psychiatric settings. Thérapie. 2017;72:427‐437. 10.1016/j.therap.2016.12.010 28336159

[30] Lisibach A , Benelli V , Ceppi MG , Waldner‐Knogler K , Csajka C , Lutters M . Quality of anticholinergic burden scales and their impact on clinical outcomes: a systematic review. Eur J Clin Pharmacol. 2021;77(2):147‐162. 10.1007/s00228-020-02994-x 33011824 PMC7803697

[31] Sumukadas D , McMurdo ME , Mangoni AA , Guthrie B . Temporal trends in anticholinergic medication prescription in older people: repeated cross‐sectional analysis of population prescribing data. Age Ageing. 2014;43(4):515‐21. 10.1093/ageing/aft199 24334709

[32] Anticholinergic Cognitive Burden Scale: 2012 Update. 2012. www.agingbraincare.org

[33] BNF . National Institute for Health and Care Excellence (NICE). Accessed February 9th 2022, 2022. https://bnf.nice.org.uk

[34] ATC/DDD Index 2022 . WHO Collaborating Centre for Drug Statistics Methodology. Updated December 14th 2021. Accessed February 9th 2022, 2022. https://www.whocc.no/atc_ddd_index/

[35] Townsend P . Deprivation. J Social Policy. 1987;16(2):125‐146. 10.1017/s0047279400020341

[36] Hanlon P , Quinn TJ , Gallacher KI , et al. Assessing Risks of Polypharmacy Involving Medications With Anticholinergic Properties. Ann Fam Med. 2020;18(2):148‐155. 10.1370/afm.2501 32152019 PMC7062487

[37] Dimitris R . Joint Models for Longitudinal and Time‐to‐Event Data With Applications in R. 1st ed. Chapman and Hall/CRC; 2012.

[38] Gray SL , Anderson ML , Dublin S , et al. Cumulative use of strong anticholinergics and incident dementia: a prospective cohort study. JAMA Intern Med. 2015;175(3):401‐7. 10.1001/jamainternmed.2014.7663 25621434 PMC4358759

[39] Diniz BS , Butters MA , Albert SM , Dew MA , Reynolds CF, 3rd . Late‐life depression and risk of vascular dementia and Alzheimer's disease: systematic review and meta‐analysis of community‐based cohort studies. Br J Psychiatry. 2013;202(5):329‐35. 10.1192/bjp.bp.112.118307 23637108 PMC3640214

[40] Fischer CE , Aguera‐Ortiz L . Psychosis and dementia: risk factor, prodrome, or cause? Int Psychogeriatr. 2018;30(2):209‐219. 10.1017/S1041610217000874 28560931

[41] Haijma SV , Van Haren N , Cahn W , Koolschijn PC , Hulshoff Pol HE , Kahn RS . Brain volumes in schizophrenia: a meta‐analysis in over 18 000 subjects. Schizophr Bull. 2013;39(5):1129‐38. 10.1093/schbul/sbs118 23042112 PMC3756785

[42] Kempton MJ. , Salvador Z , Munafo MR. , et al. Structural neuroimaging studies in major depressive disorder. Meta analysis and comparison with bipolar disorder. Archives of General Psychiatry. 2011;68(7):675‐690. 10.1001/archgenpsychiatry.2011.60 21727252

[43] Sen A , Capelli V , Husain M . Cognition and dementia in older patients with epilepsy. Brain. 2018;141(6):1592‐1608. 10.1093/brain/awy022 29506031 PMC5972564

[44] Wilkinson T , Schnier C , Bush K , et al. Identifying dementia outcomes in UK Biobank: a validation study of primary care, hospital admissions and mortality data. Eur J Epidemiol. 2019;34(6):557‐565. 10.1007/s10654-019-00499-1 30806901 PMC6497624

[45] Fry A , Littlejohns TJ , Sudlow C , et al. Comparison of sociodemographic and health‐related characteristics of UK Biobank Participants with those of the general Population. Am J Epidemiol. 2017;186(9):1026‐1034. 10.1093/aje/kwx246 28641372 PMC5860371

[46] Andrade C . Anticholinergic drug exposure and the risk of dementia: there is modest evidence for an association but not for causality. J Clin Psychiatry. 2019;80(4) 10.4088/JCP.19f13000 31390497

[47] Kersten H , Wyller TB . Anticholinergic drug burden in older people's brain ‐ how well is it measured? Basic Clin Pharmacol Toxicol. 2014;114(2):151‐9. 10.1111/bcpt.12140 24112192

[48] Lozano‐Ortega G , Johnston KM , Cheung A , et al. A review of published anticholinergic scales and measures and their applicability in database analyses. Arch Gerontol Geriatr. 2020;87:103885. 10.1016/j.archger.2019.05.010 31155228

[49] Salahudeen MS , Duffull SB , Nishtala PS . Anticholinergic burden quantified by anticholinergic risk scales and adverse outcomes in older people: a systematic review. BMC Geriatr. 2015;15:31. 10.1186/s12877-015-0029-9 25879993 PMC4377853

[50] Salahudeen MS , Hilmer SN , Nishtala PS . Comparison of anticholinergic risk scales and associations with adverse health outcomes in older people. J Am Geriatr Soc. 2015;63(1):85‐90. 10.1111/jgs.13206 25597560

