# Supplementary material for: Association between anticholinergic burden and dementia in UK Biobank
Source: Alzheimers Dement (N Y). 2022 Apr 12;8(1):e12290. doi: 10.1002/trc2.12290 (PMC9005668; doi:10.1002/trc2.12290)
Supplement: Supplementary file 1 — Supporting Information [file TRC2-8-e12290-s003.docx]

**Supplementary Figure 1**: The data cleaning procedure. The grey boxes contain the number of prescriptions (top row in the boxes) and participants (bottom row in the boxes) when a unit of observation was a single prescription; the blue boxes contain the number of prescriptions/participants (both take the same value) when the data was formatted so that the unit of observation was the yearly AChB for a participant in year 0. The orange ellipses contain numbers of prescriptions (top row in the ellipses) and participants (bottom row in the ellipses) that were removed at each data-cleaning step. The data cleaning steps include: (1) removal of prescription entries that were blank (i.e., did not list a drug), (2) removal of prescriptions without dates or with invalid dates, (3) the “separation“ of prescriptions with multiple anticholinergic compounds into single entries, (4) removal of prescriptions occurring after the recorded dates of death, (5) removal of prescriptions in years other than year 0, (6) removal of participants diagnoses with dementia prior to year 0 or within one year of year 0, (7) removal of participants diagnosed with Parkinson’s disease, Huntington’s disease, Creutzfeldt-Jacob disease, or multiple sclerosis, (8) removal of participants younger than 60 at the end of sampling or when diagnosed with dementia, (9) removal of participants for whom year 0 was prior to 2015. Please note that in the third step, when prescriptions were “separated” so that prescriptions originally containing several anticholinergic compounds were divided into separate prescriptions (with a single anticholinergic compound each), the number of observations in the dataset effectively *increased*.


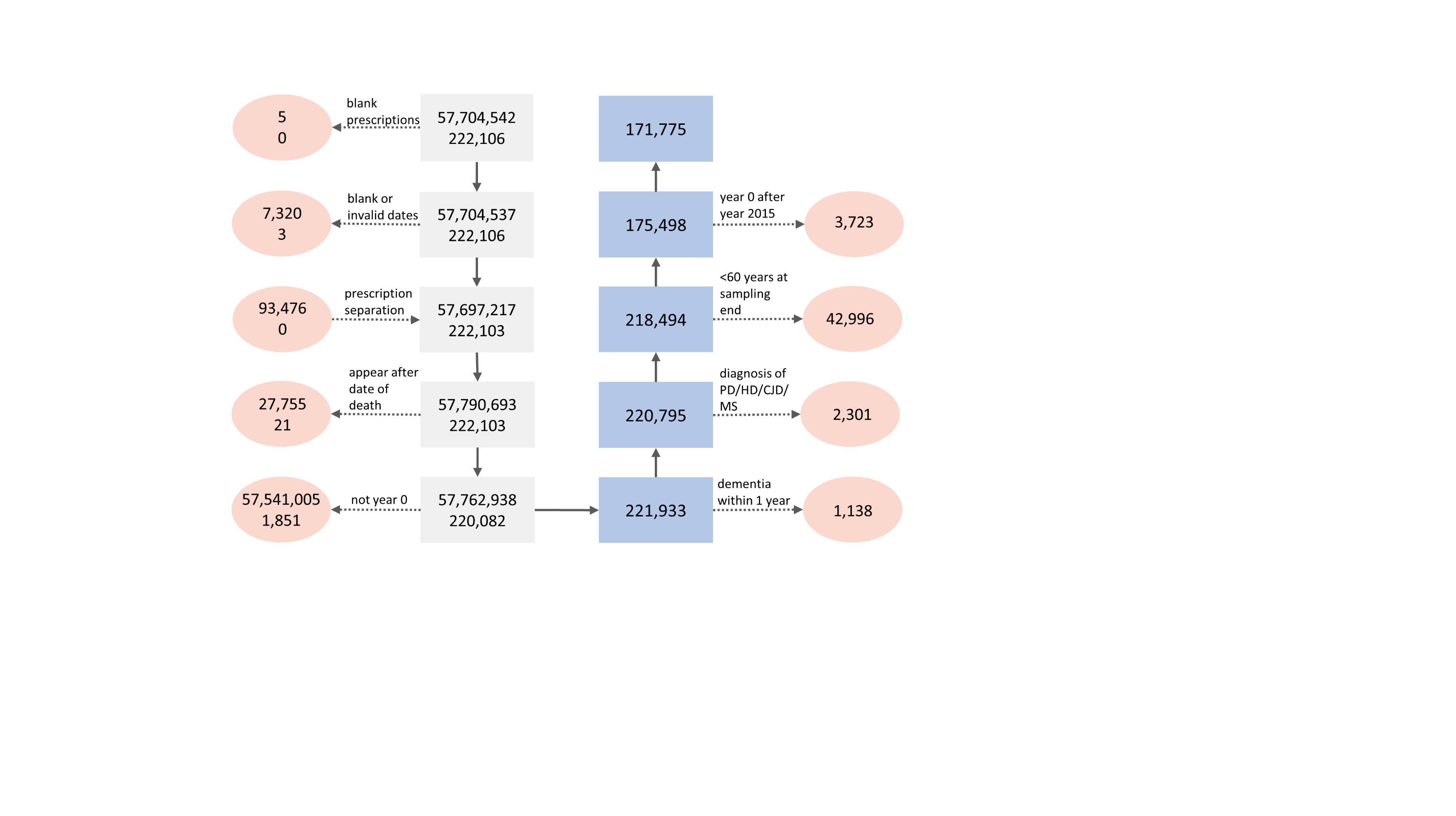


**Supplementary Figure 2**: Martingale residuals plotted against individual continuous covariates. Depicted are only those covariates for which this relationship was judged not to be linear before transforming. For each covariate, four plots are depicted, where the covariate is either untransformed (top left), or square-root-, log- , or rank-based-inverse-normal transformed.


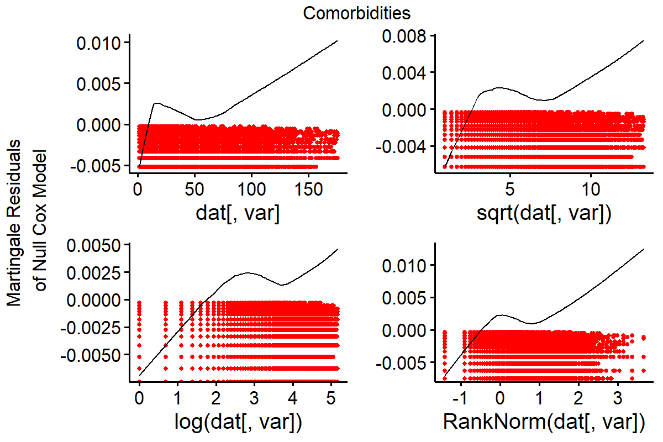

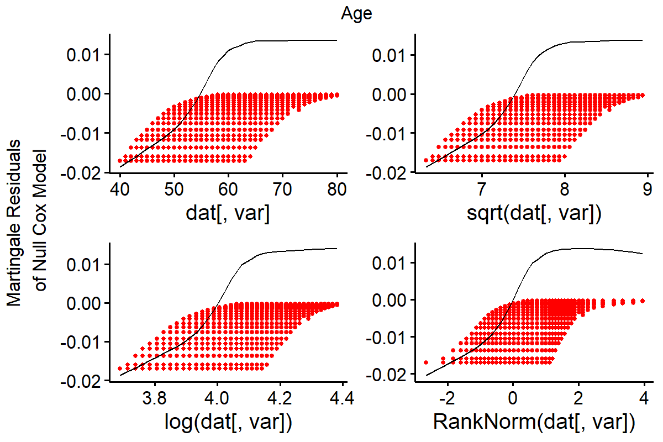

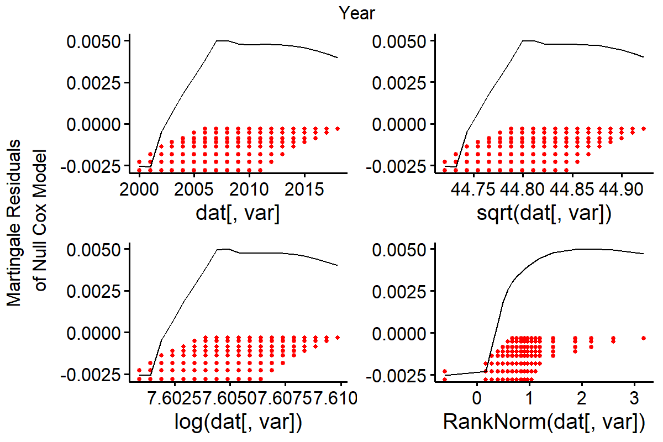

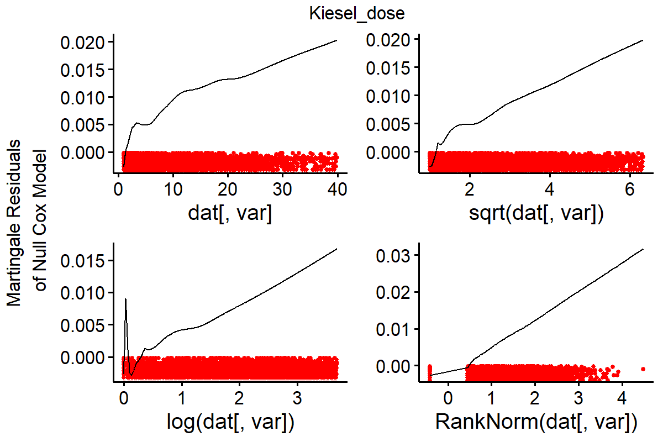

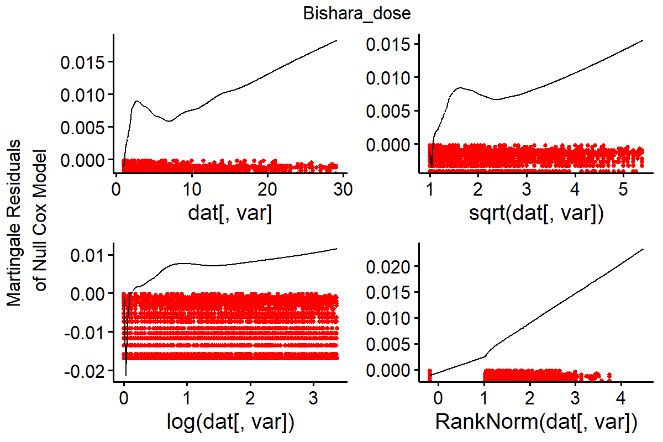

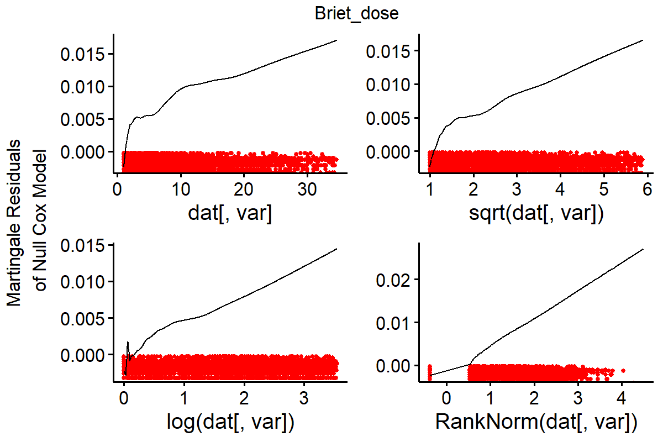

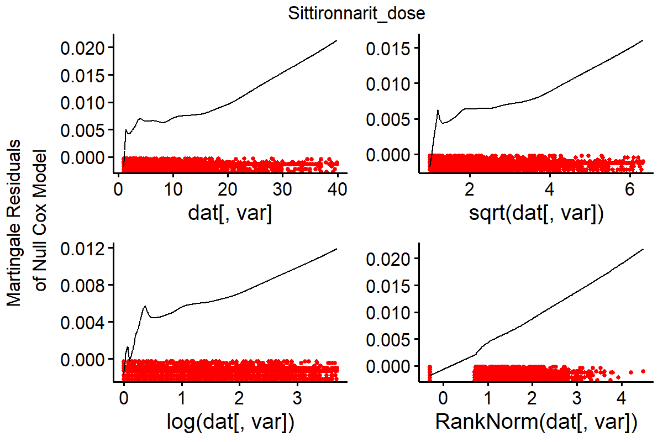

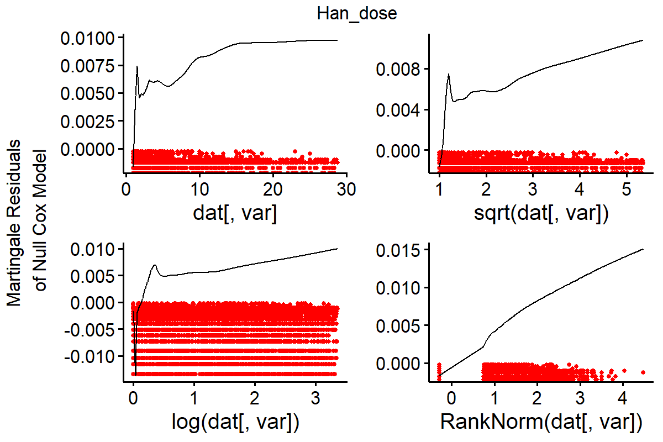

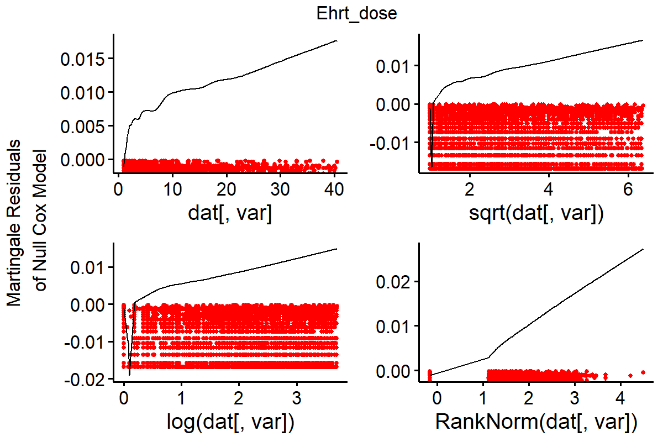

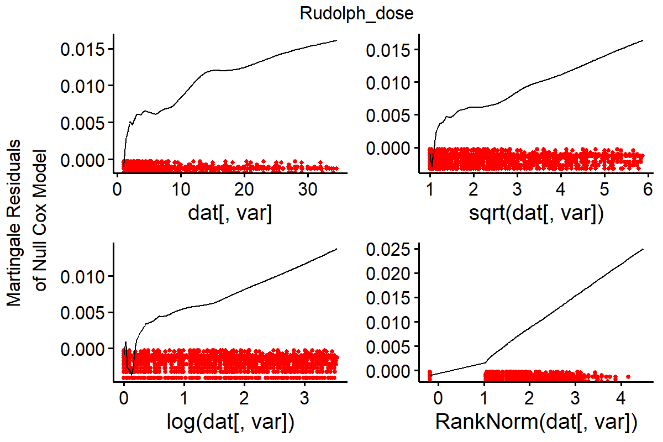

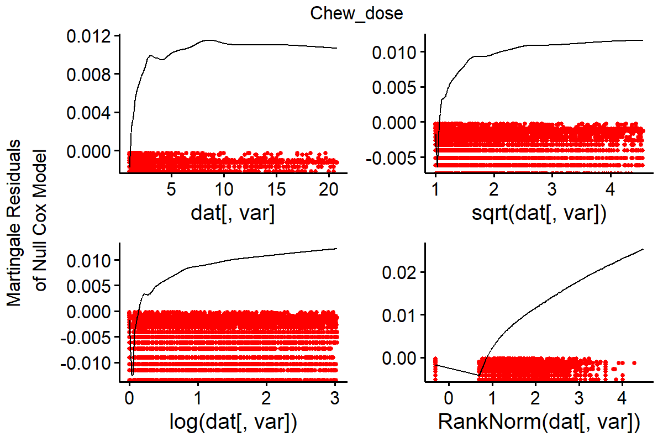

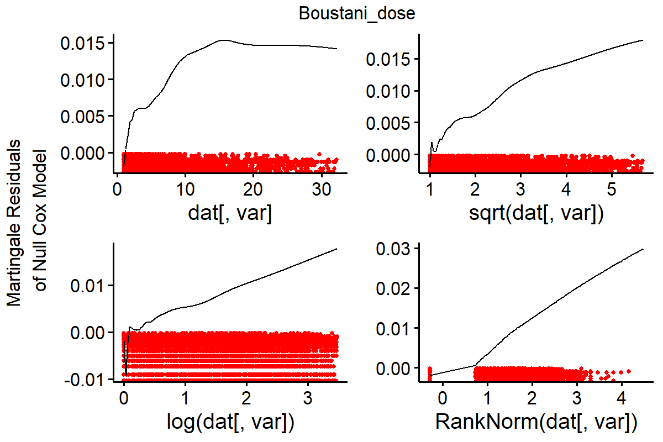

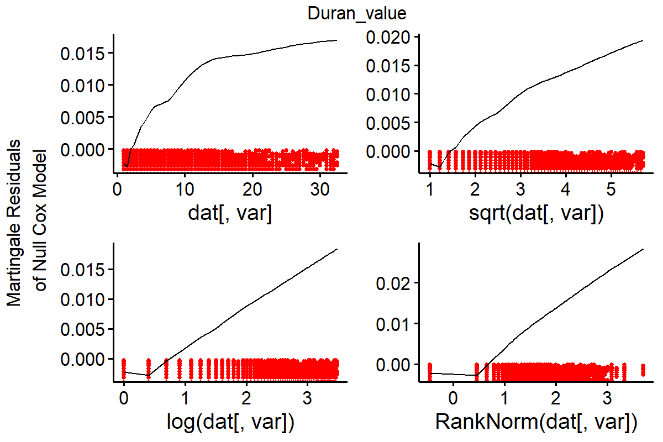

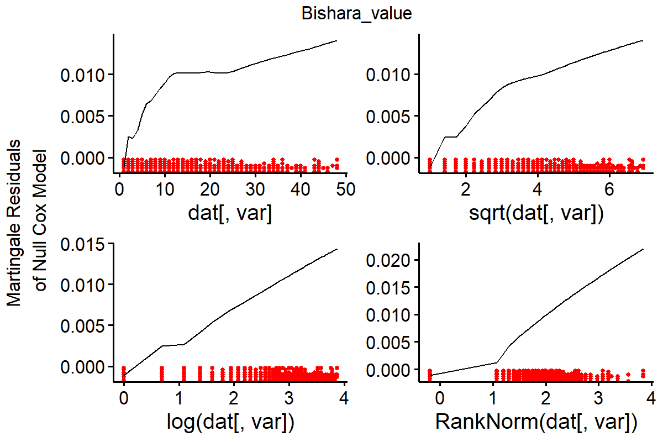

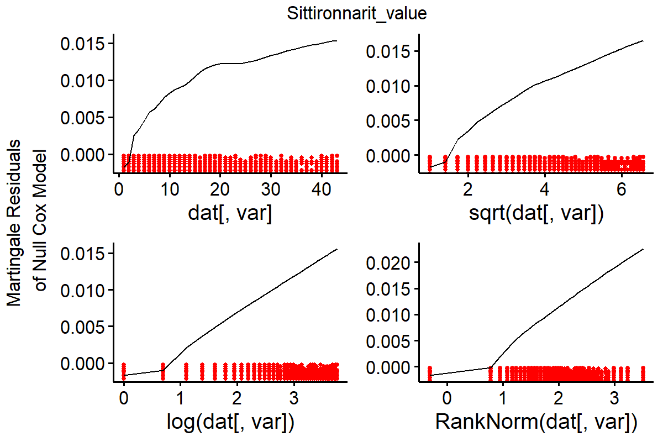

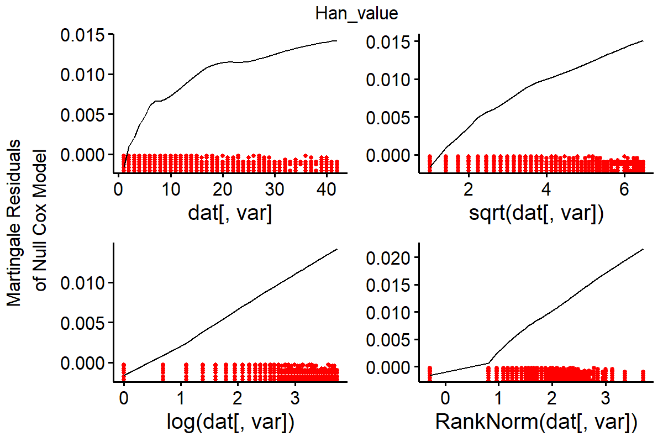

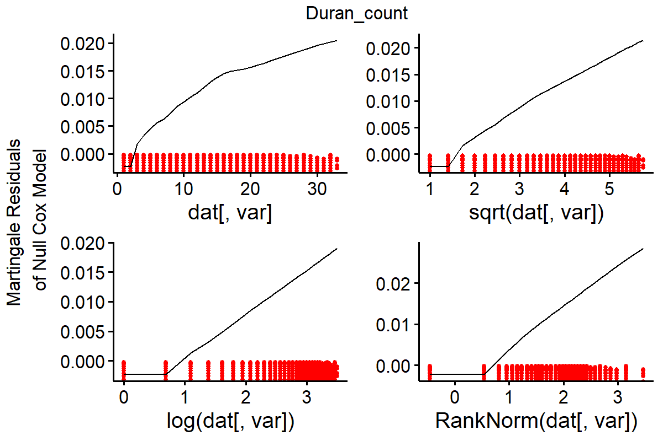

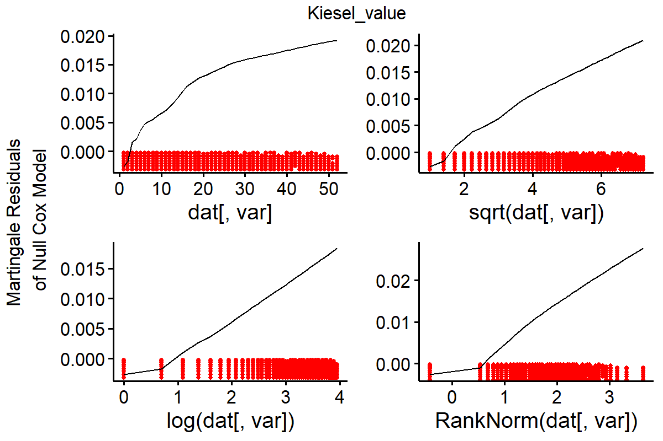

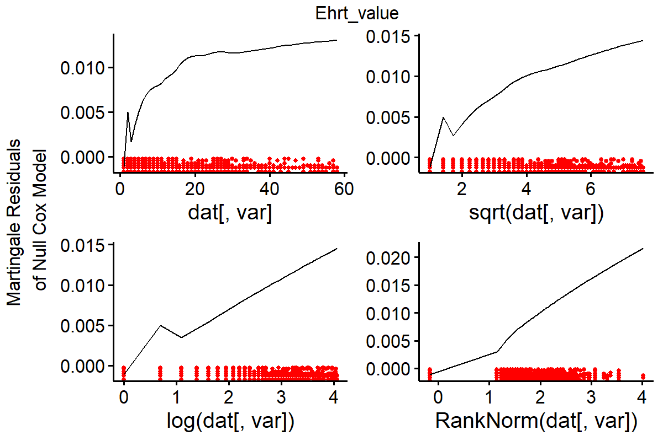

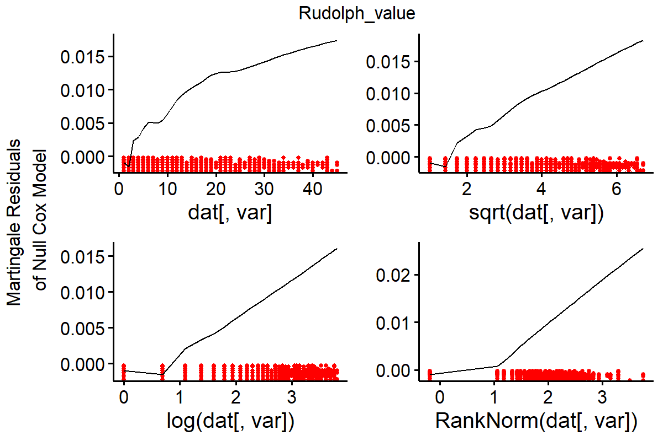

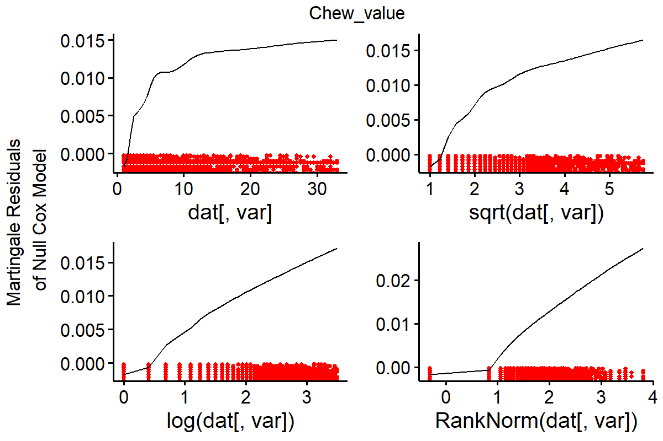

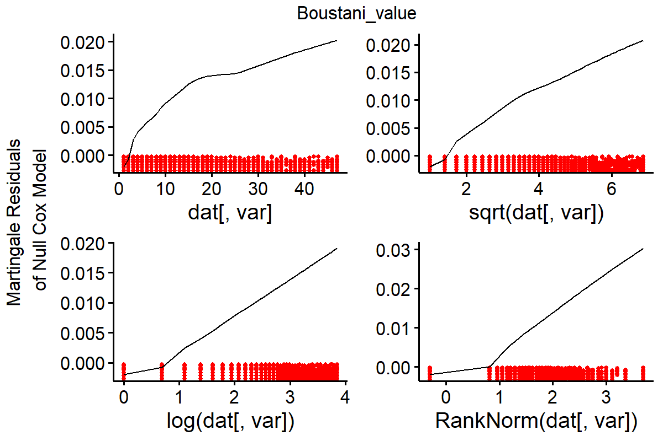

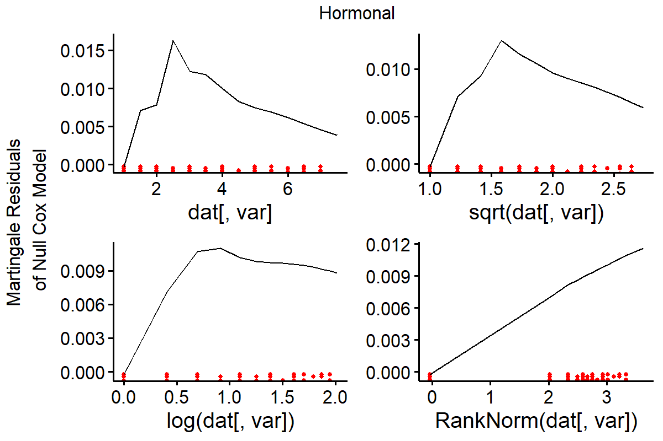

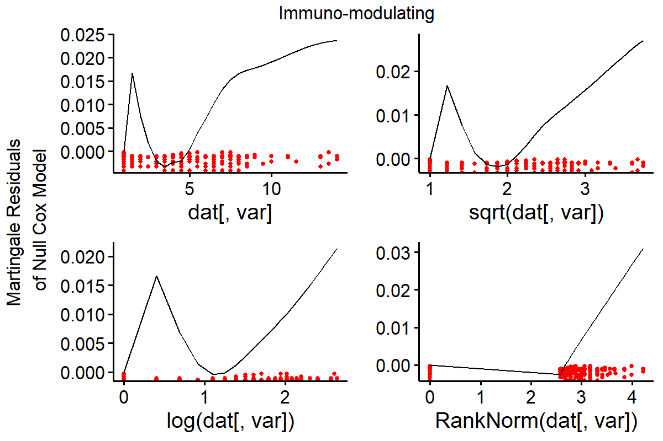

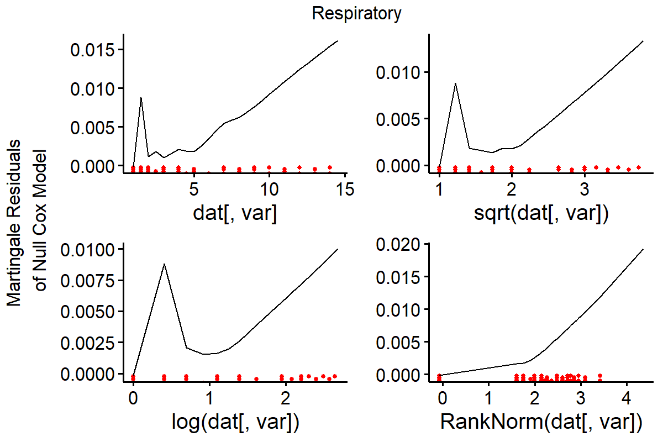

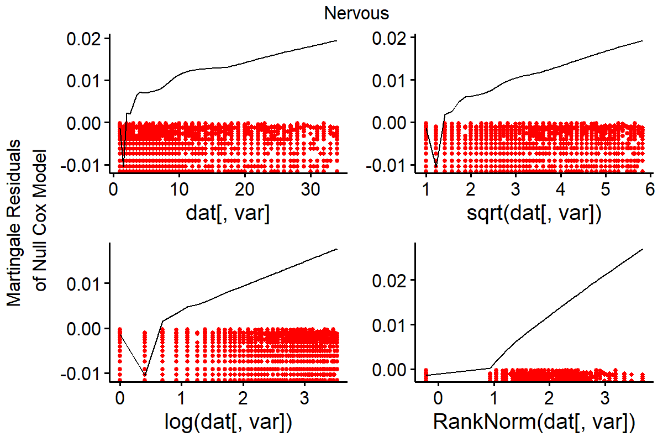

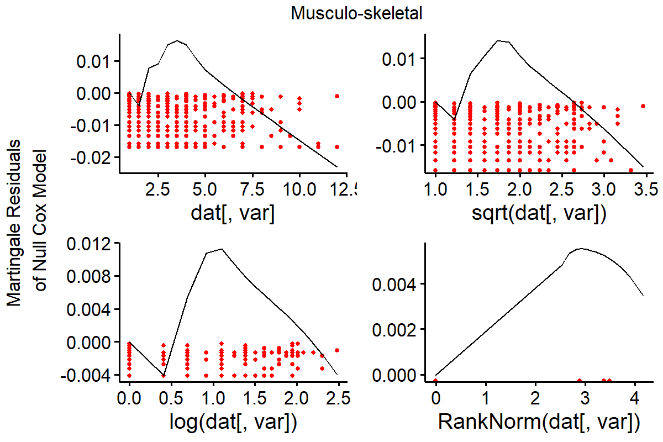

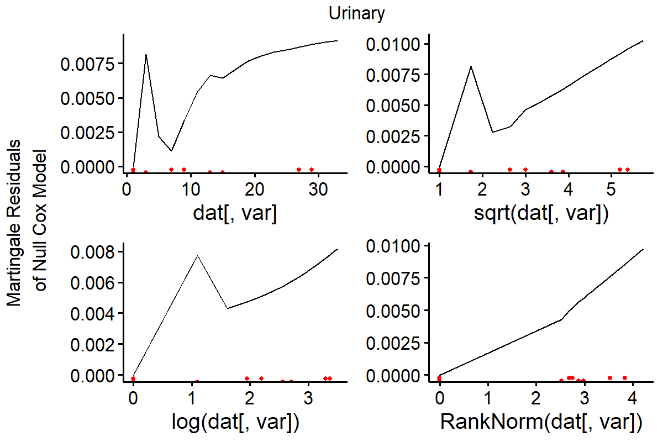

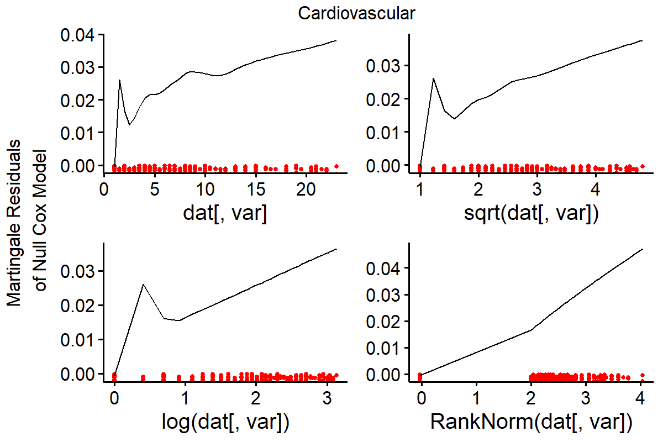

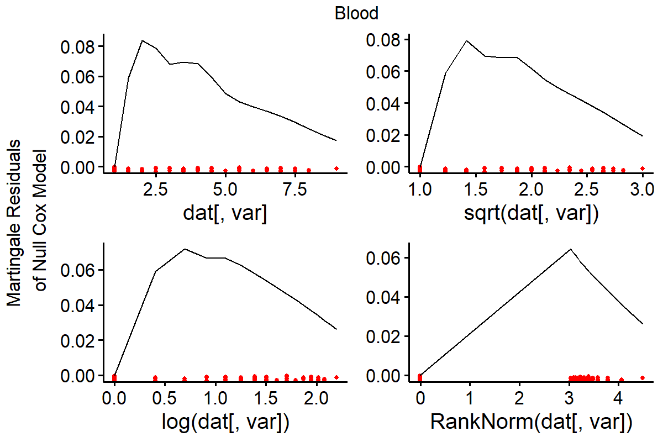

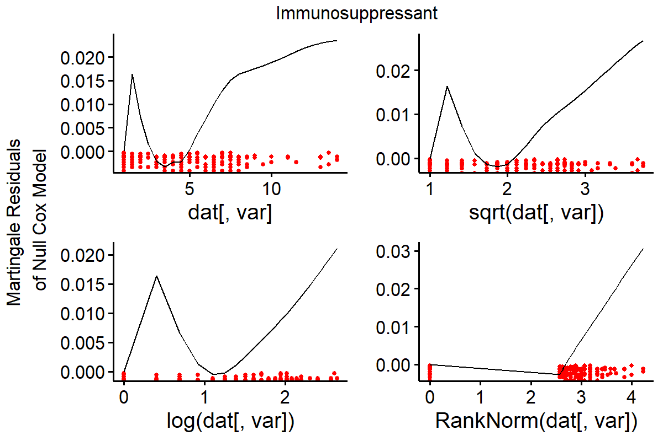

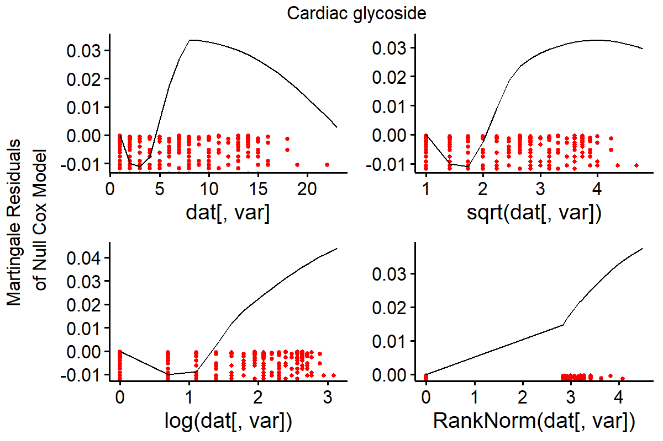

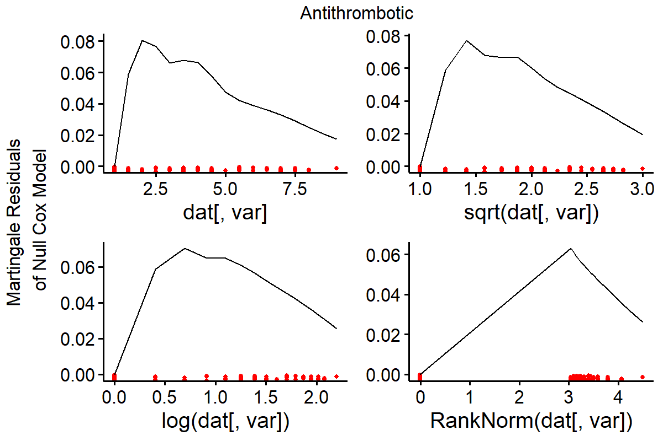

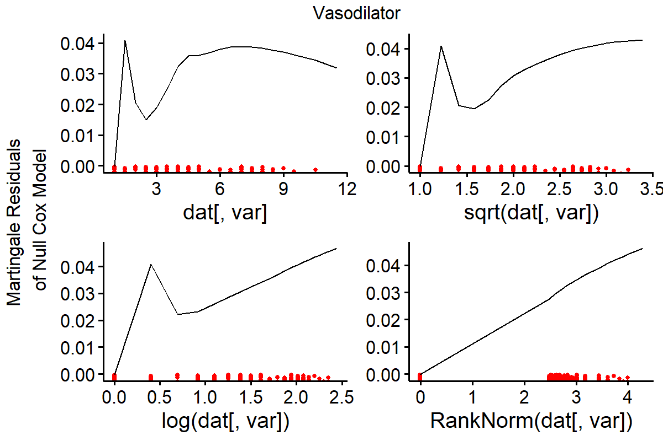

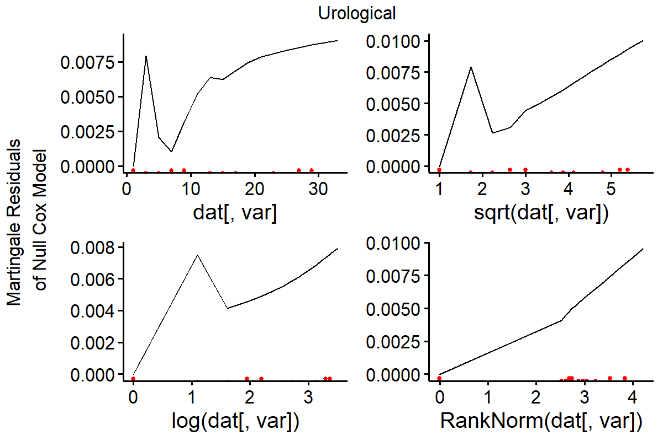

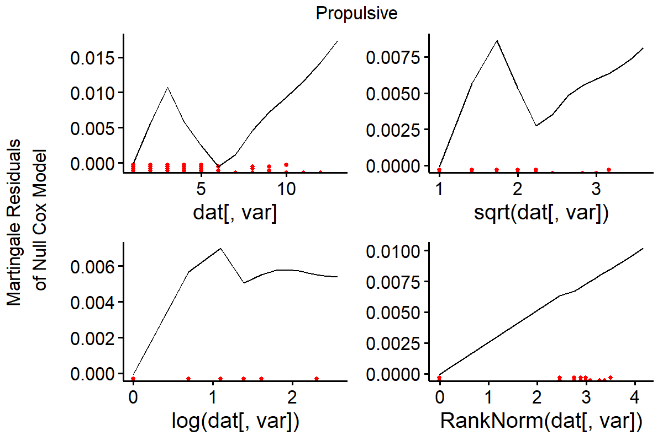

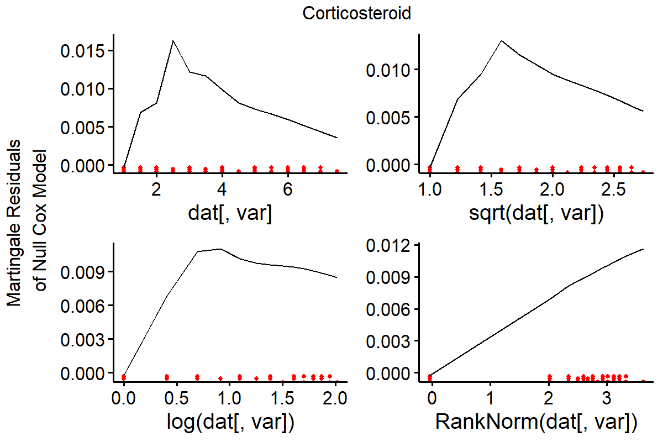

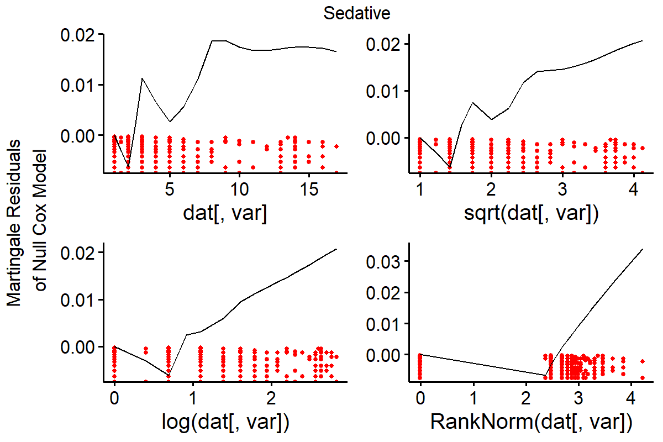

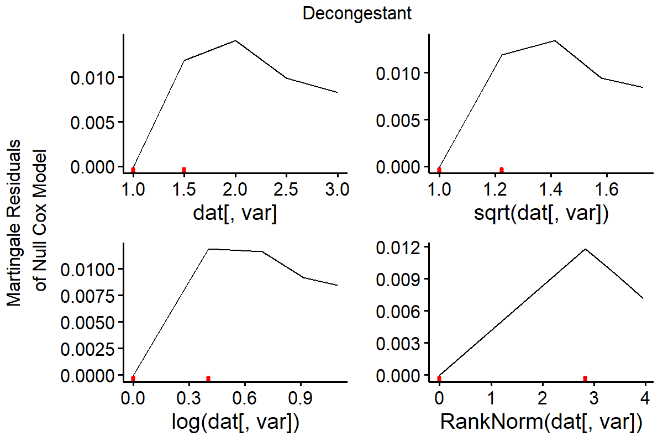

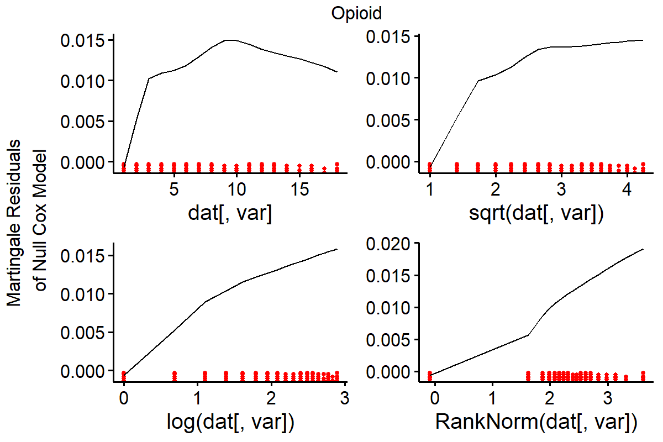

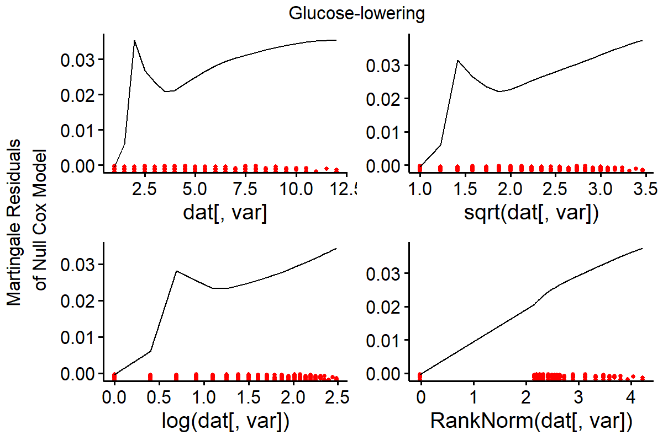

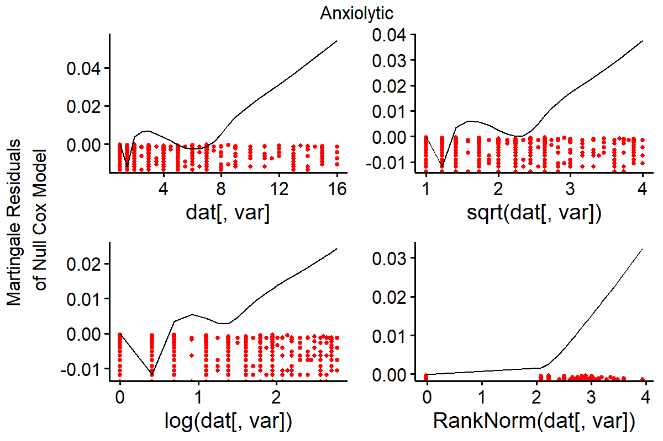

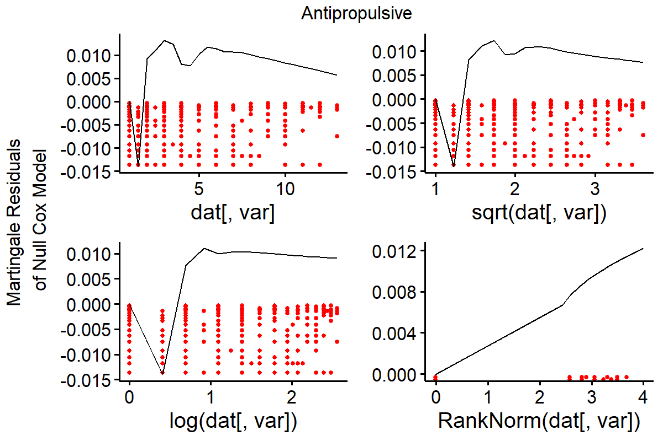

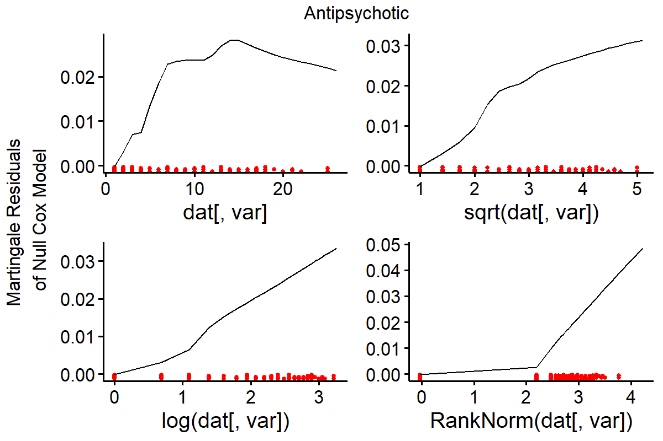

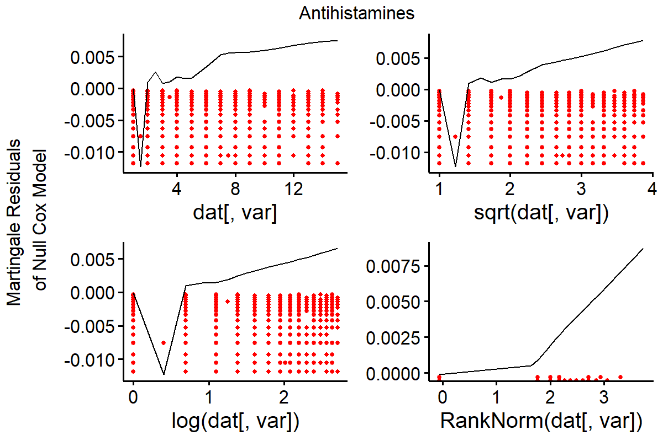

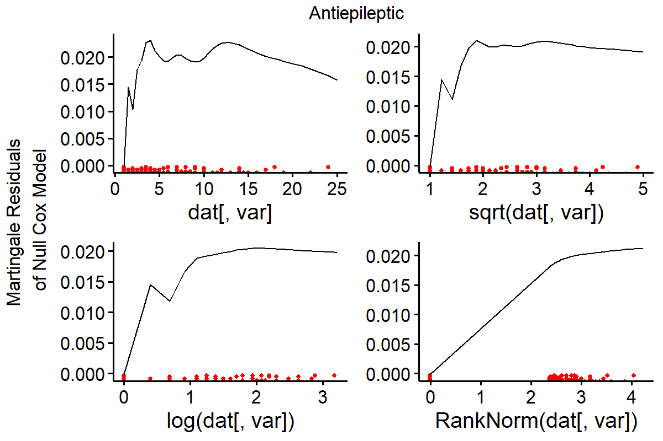

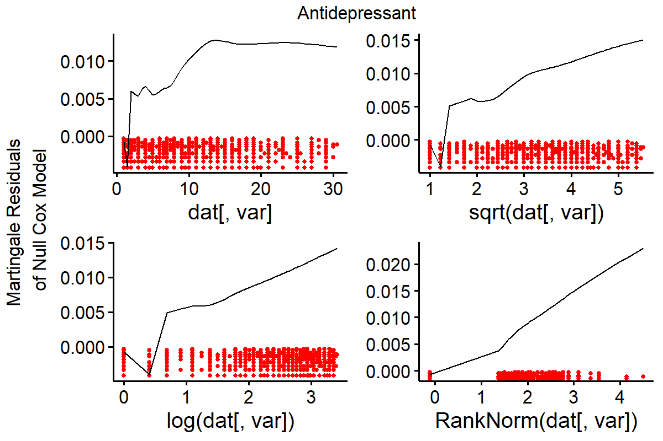

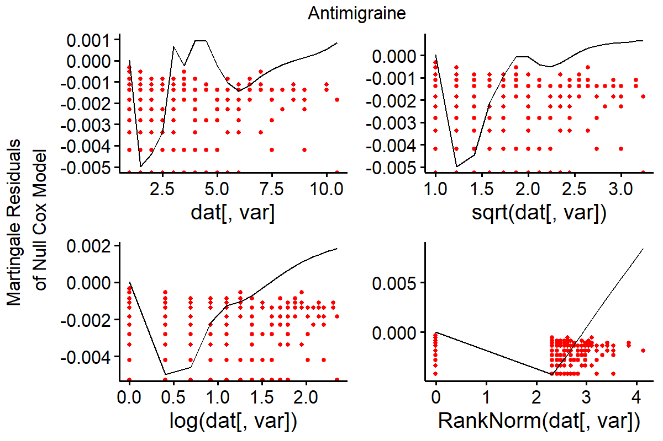


**Supplementary Figure 3**: HRs for the association between AChB and dementia (top panels) and drug count and dementia (bottom panels) when AChB is scaled (**top row**), log-transformed and scaled (**middle row**), and transformed using the rank-inverse normal transformation and scaled (**bottom row**). The colour indicates the anticholinergic scales used for the calculation of the AChB; the symbols and line types indicate the type of scale computation used. The left and right columns show uncorrected results and results corrected for multiple testing, respectively.


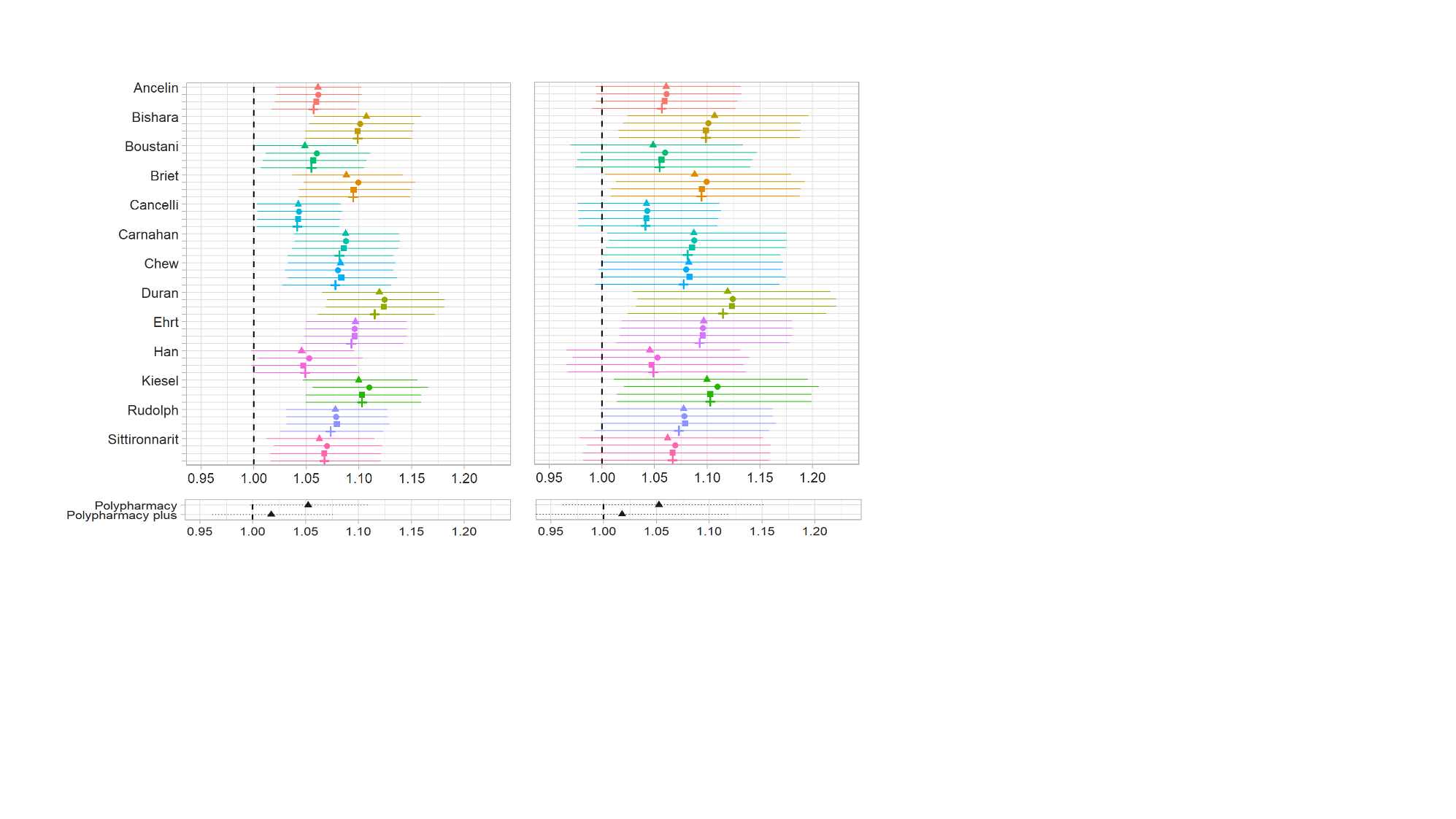

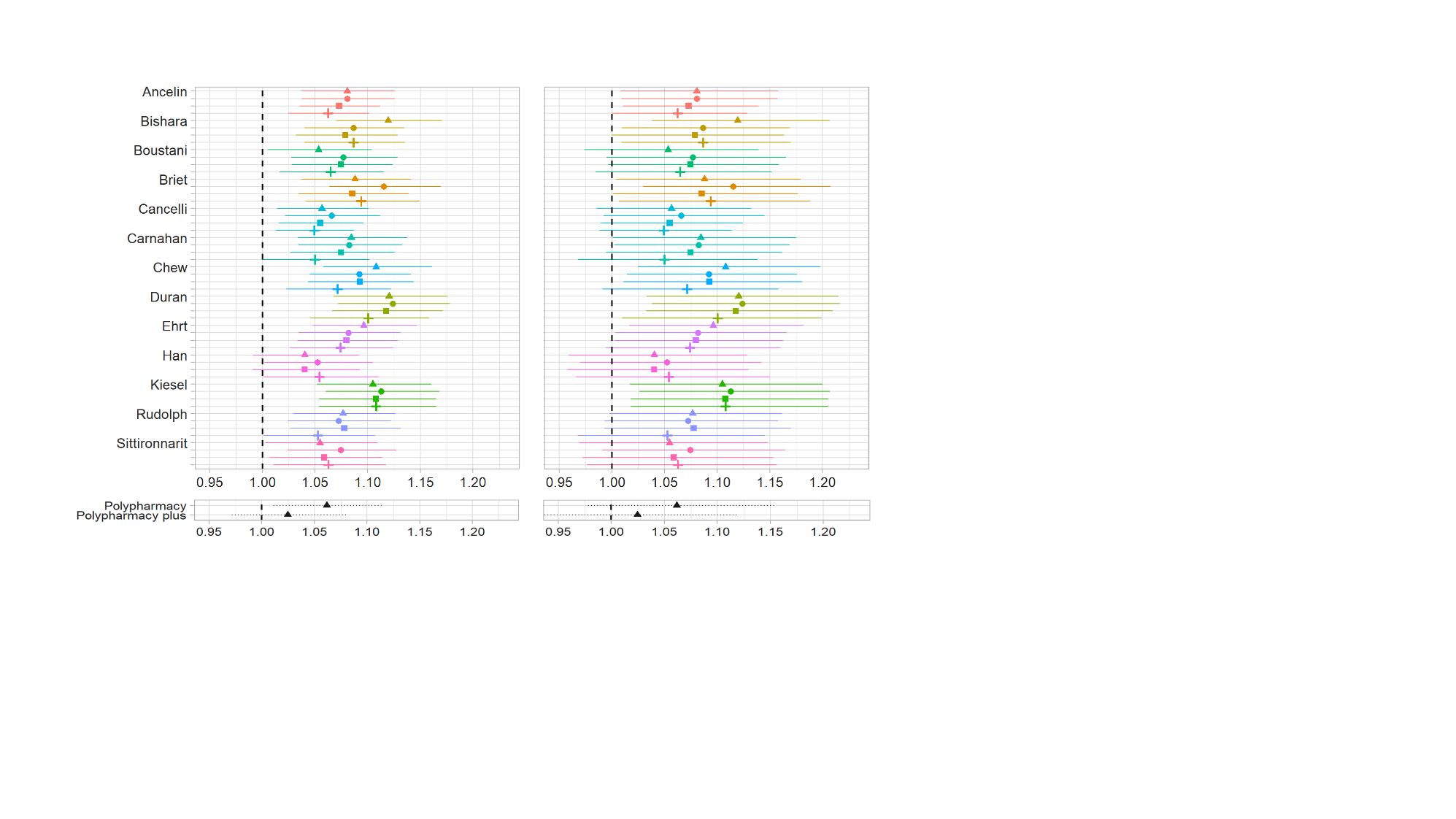

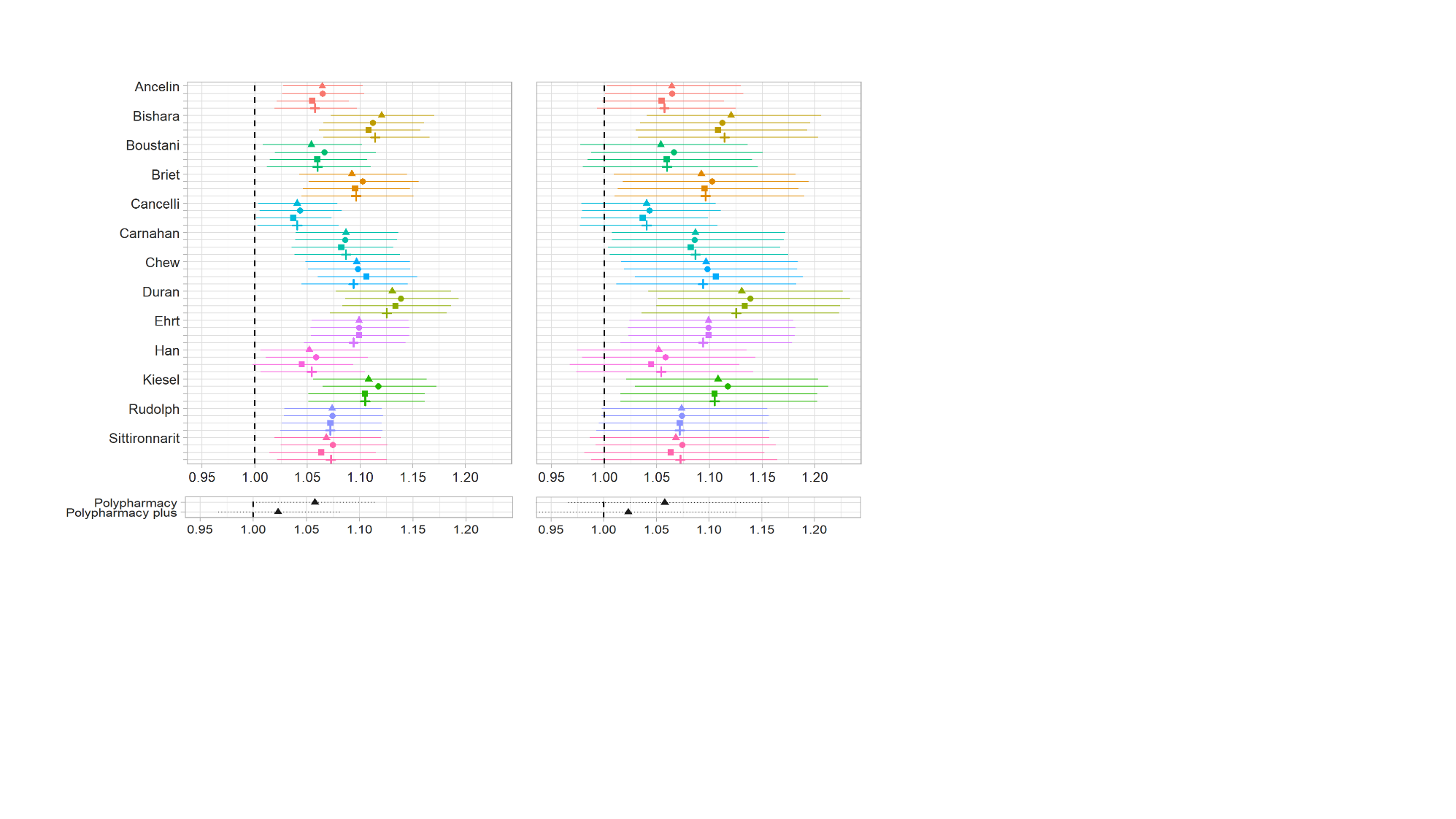

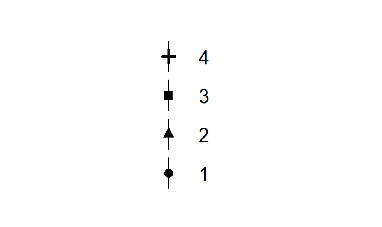


**Scale type**

**dosage/quantity-based**

**dosage-based

count-based

value-based**

**
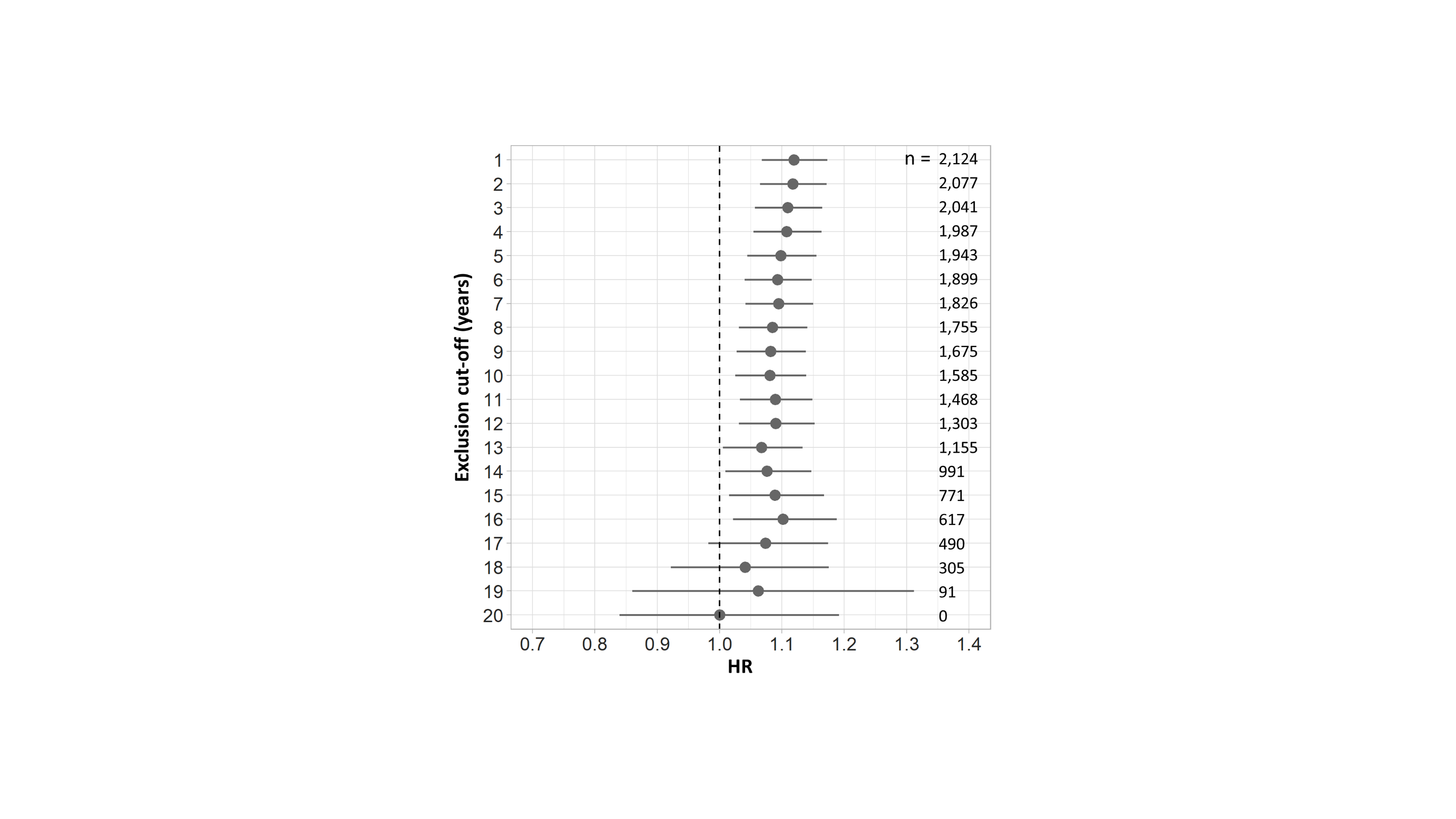
Supplementary** **Figure 4**: Association between AChB according to Dúran et al. (2013) and dementia, with different exclusion cut-offs for period of dementia diagnosis. Participants in the sample were diagnosed with dementia at different time points after year 0. If the diagnosis for a participant was established within a certain cut-off period after year 0, that participant was removed from the sample before analysis. The numbers on the y-axis specify this cut-off period (years after year 0); the x-axis represents the effect size (HR). The numbers on the right indicate the numbers of dementia cases for each model: as the exclusion period increases, more participant get excluded and the sample size decreases.

**Supplementary** **Table 2**: Anticholinergics scales identified in the present study. We considered anticholinergic scales that were available as complete lists of drugs, scored each drug for its anticholinergic potency, and did not utilize dosage. Grey shading indicates that the scale was not considered for further analysis. For two scales^1,2^, updated versions were used (Aging Brain Care, 2012; Carnahan, 2014, personal communication on 21.10.2019). One scale^3^ was modified to include newer drugs from the UK market as has been done before^4^. Some drugs from one scale^5^ were categorised as “drugs with improbable or no anticholinergic action”. For our analyses, the drugs in that category were scored with 0.5. Modified from Mur et al. (**IN PRESS**).

| **Surname of first author** | **Scale name** | **Year of publication** | **Reason for exclusion** |
| --- | --- | --- | --- |
| Summers^6^ | Drug Risk Number (DRN) | 1978 | Outdated (based on the date of publication and on new scales developed on its basis). |
| Han^7,8^ | Clinician-rated Anticholinergic Scale (CrAS) | 2001 |  |
| Aizenberg^9^ | Anticholinergic Burden Score (ABS) | 2002 | Publicly unavailable and no response from lead author to two email requests within a year. |
| Minzenberg^10^ | n.a. | 2004 | Based on a reference compound. |
| Ancelin^11^ | Anticholinergic Burden Classification (ABC) scale | 2006 |  |
| Carnahan^1^ | Anticholinergic Drug Scale (ADS) | 2006 |  |
| Hilmer^12^ | Drug Burden Index (DBI) | 2007 | Required information on drug dosage. |
| Chew^13^ | Anticholinergic Activity Scale (AAS) | 2008 |  |
| Cancelli^14^ | n.a. | 2008 |  |
| Rudolph^3^ | Anticholinergic Risk Scale (ARS) | 2008 |  |
| Ehrt^15^ | Revised Anticholinergic Activity Scale (AAS-r) | 2010 |  |
| Sittironnarit^16^ | Anticholinergic Loading Scale (ALS) | 2011 |  |
| Boustani^2^ | Anticholinergic Cognitive Burden (ACB) | 2008 |  |
| Whalley^17^ | n.a. | 2012 | Unavailable in full. |
| Durán^5^ | n.a. | 2013 |  |
| Faure^18^ | Drug Burden Index, International Version (DBI-WHO) | 2014 | Required information on drug dosage. |
| Klamer^19^ | MARANTE | 2017 | Required information on drug dosage. |
| Bishara^20^ | Anticholinergic effect on cognition (AEC) scale | 2017 |  |
| Briet^21^ | Anticholinergic impregnation scale | 2017 |  |
| Kiesel^22^ | n.a. | 2018 |  |

**Supplementary** **Table 4**: Numbers of participants diagnosed with dementia in different groups of the sample.

| **Group** | **N** | **N cases (% of group)** |
| --- | --- | --- |
| All | 171,775 | 2,124 (1.2) |
| Sex | | |
| Male | 77,465 | 1,139 (1.5) |
| Female | 94,310 | 985 (1.0) |
| Age group | | |
| 60-64 | 34,050 | 33 (0.10) |
| 65-69 | 37,306 | 149 (0.40) |
| 70-74 | 51,436 | 534 (1.04) |
| 75-79 | 41,810 | 1,134 (2.7) |
| >= 80 | 7,173 | 274 (3.8) |

**Supplementary Table 5**: Descriptive statistics of variables used in the models, presented separately for participants diagnosed with dementia and those not diagnosed with dementia. Data providers are suppliers of computer systems for general practitioners in the UK. The provide the IT framework for general practice surgeries to store information on patients, diagnoses, and prescriptions. The division of data providers in the UK is partially determined by the region in which a general practice surgery is located: England, Scotland, or Wales. *The total number of prescriptions was used along the number of anticholinergic drugs to calculate the scale-specific non-anticholinergic drug count.

|  |  | **Dementia diagnosis** | **No dementia diagnosis** |
| --- | --- | --- | --- |
| Participant count | | 2,124 | 169,631 |
| **Variable** | **Level** | **Median (IQR) or n (%)** | |
| Age |  | 59 (5) | 54 (10) |
| Sex | Female | 985 (46.4) | 93,325 (55.0) |
| Education | No graduate degree | 1,672 (80.5) | 116,519 (69.6) |
| Deprivation |  | -1.96 (4.7) | -2.30 (3.8) |
| Alcohol consumption | Daily or almost daily Three or four times a week Once or twice a week Once to three times a month Only special occasions Never | 475 (22.5) 382 (18.1)  461 (21.8) 190 (9.0)  317 (15.0) 289 (13.7) | 35,514 (21.0) 39,365 (23.3)  43,354 (25.6) 17,959 (10.6)  19,356 (11.4) 13,735 (8.1) |
| Smoking | Current smoker  Previous smoker  Non-smoker | 234 (11.1) 935 (44.4) 935 (44.4) | 16,178 (9.6) 62,437 (37.0) 90,156 (53.4) |
| Physical activity | Strenuous  Moderate  Light | 80 (4.3) 1105 (59.9) 661 (35.8) | 13,497 (8.6) 102,016 (64.7) 42,116 (26.7) |
| BMI | <18.5 18.5-25 25-30 30-35 35-40 >40 | 13 (0.62) 606 (28.8) 877 (41.7) 431 (20.5) 129 (6.1) 45 (2.1) | 755 (0.45) 51,043 (30.2) 73,315 (43.4) 31,376 (18.6) 8,941 (5.3) 3,327 (2.0) |
| Data provider | England (Vision) Scotland England (TPP) Wales | 150 (7.1) 40 (1.9) 1,852 (87.2) 82 (3.9) | 13,886 (8.2) 18,718 (11.0) 121,281 (71.5) 15,766 (9.3) |
| Prior depression |  | 257 (12.1) | 12,879 (7.6) |
| Prior stroke |  | 66 (3.1) | 1,532 (0.90) |
| Prior diabetes |  | 184 (8.7) | 3,850 (2.3) |
| Prior hypercholesterolemia |  | 145 (6.8) | 4,756 (2.8) |
| Prior hypertension |  | 415 (19.5) | 15,737 (9.3) |
| Number of prior comorbidities |  | 27 (44) | 18 (40) |
| Total number of prescriptions* |  | 7 (19) | 3 (12) |
| *APOE* carrier | ε2  ε3  ε4 | 191 (7.8) 920 (44.6) 981 (47.6) | 21,465 (13.0) 101,820 (61.5) 41,218 (25.5) |

**Supplementary Table 6**: Frequency of anticholinergic prescribing in the sample from 2000 to 2015 and in year 0 according to each anticholinergic scale studied.

| **Scale** | **Number of distinct anticholinergic drugs in the sample** | **Number of anticholinergic drugs  (% of prescriptions)** | **Number of distinct anticholinergic drugs in the sample in year 0** | **Number of anticholinergic drugs in year 0 (% of prescriptions)** | **Anticholinergic prescriptions per person in year 0** |
| --- | --- | --- | --- | --- | --- |
| Ancelin | 21 | 1,086,739 (2.5) | 20 | 42,068 (2.6) | 0.24 |
| Bishara | 58 | 2,876,150 (6.6) | 56 | 126,195 (7.9) | 0.72 |
| Boustani | 90 | 5,272,868 (12.2) | 87 | 225,409 (14.1) | 1.28 |
| Briet | 121 | 7,041,395 (16.3) | 117 | 294,881 (18.4) | 1.68 |
| Cancelli | 14 | 1,700,948 (3.9) | 13 | 63,609 (4.0) | 0.36 |
| Carnahan | 111 | 3,761,500 (8.7) | 105 | 165,838 (10.4) | 0.94 |
| Chew | 36 | 4,739,876 (11.0) | 34 | 177,101 (11.1) | 1.00 |
| Durán | 147 | 8,257,133 (19.1) | 139 | 320,792 (20.0) | 1.83 |
| Ehrt | 24 | 3,079,302 (7.1) | 23 | 119,600 (7.5) | 0.68 |
| Han | 54 | 4,378,190 (10.1) | 52 | 193,108 (12.1) | 1.10 |
| Kiesel | 141 | 9,495,193 (22.0) | 136 | 371,757 (23.2) | 2.12 |
| Rudolph | 61 | 2,201,774 (5.1) | 59 | 102,540 (6.4) | 0.58 |
| Sittironnarit | 47 | 5,129,912 (11.9) | 46 | 207,085 (12.9) | 1.18 |

**Supplementary Table 7** (see also next page): HRs for scaled (mean=0; standard deviation=1) numerical variables in the Cox proportional risks model predicting the risk of dementia. Each row depicts the effect of anticholinergic burden according to a different anticholinergic scale. The different columns depict HRs for different transformations of the data.

| **Scale** | **Type** | **Untransformed** | | **Log** | | **Rank-based inverse-normal** | | **n missing** | |
| --- | --- | --- | --- | --- | --- | --- | --- | --- | --- |
|  |  | **HR** | **99.9% CI** | **HR** | **99.9% CI** | **HR** | **99.9% CI** |  |  |
| Ancelin | count | 1.08 | 1.01 - 1.16 | 1.06 | 1.00 - 1.13 | 1.07 | 0.99 - 1.13 | 23,367 |  |
|  | value | 1.08 | 1.01 - 1.16 | 1.06 | 1.00 - 1.13 | 1.07 | 0.99 - 1.13 | 23,371 |  |
|  | dosage | 1.07 | 1.01 - 1.14 | 1.05 | 1.00 - 1.11 | 1.07 | 0.99 - 1.13 | 23,934 |  |
|  | quantity | 1.06 | 1.00 - 1.13 | 1.06 | 0.99 - 1.12 | 1.06 | 0.99 - 1.13 | 29,712 |  |
| Bishara | count | 1.12 | 1.04 - 1.21 | 1.12 | 1.04 - 1.21 | 1.07 | 1.02 - 1.20 | 23,472 |  |
|  | value | 1.09 | 1.01 - 1.17 | 1.11 | 1.03 - 1.20 | 1.08 | 1.02 - 1.19 | 23,553 |  |
|  | dosage | 1.08 | 1.00 - 1.16 | 1.11 | 1.03 - 1.19 | 1.08 | 1.02 - 1.19 | 24,130 |  |
|  | quantity | 1.09 | 1.01 - 1.17 | 1.11 | 1.03 - 1.20 | 1.08 | 1.02 - 1.19 | 29,826 |  |
| Boustani | count | 1.05 | 0.97 - 1.14 | 1.05 | 0.98 - 1.14 | 1.10 | 0.97 - 1.13 | 23,525 |  |
|  | value | 1.08 | 0.99 - 1.17 | 1.07 | 0.99 - 1.15 | 1.10 | 0.98 - 1.15 | 23,698 |  |
|  | dosage | 1.07 | 1.00 - 1.16 | 1.06 | 0.98 - 1.14 | 1.11 | 0.98 - 1.14 | 24,424 |  |
|  | quantity | 1.06 | 0.98 - 1.15 | 1.06 | 0.98 - 1.15 | 1.10 | 0.98 - 1.14 | 29,859 |  |
| Briet | count | 1.09 | 1.00 - 1.18 | 1.09 | 1.01 - 1.18 | 1.05 | 1.00 - 1.18 | 23,606 |  |
|  | value | 1.12 | 1.03 - 1.21 | 1.10 | 1.02 - 1.19 | 1.05 | 1.01 - 1.19 | 23,784 |  |
|  | dosage | 1.09 | 1.00 - 1.18 | 1.10 | 1.01 - 1.18 | 1.05 | 1.01 - 1.19 | 24,327 |  |
|  | quantity | 1.09 | 1.01 - 1.19 | 1.10 | 1.01 - 1.19 | 1.05 | 1.01 - 1.19 | 29,935 |  |
| Cancelli | count | 1.06 | 0.99 - 1.13 | 1.04 | 0.98 - 1.11 | 1.09 | 0.98 - 1.11 | 23,387 |  |
|  | value | 1.07 | 0.99 - 1.15 | 1.04 | 0.98 - 1.11 | 1.10 | 0.98 - 1.11 | 23,399 |  |
|  | dosage | 1.05 | 0.99 - 1.12 | 1.04 | 0.98 - 1.10 | 1.10 | 0.98 - 1.11 | 23,966 |  |
|  | quantity | 1.05 | 0.99 - 1.11 | 1.04 | 0.98 - 1.11 | 1.10 | 0.98 - 1.11 | 29,730 |  |
| Carnahan | count | 1.08 | 1.00 - 1.18 | 1.09 | 1.01 - 1.17 | 1.11 | 1.01 - 1.18 | 23,464 |  |
|  | value | 1.08 | 1.00 - 1.17 | 1.09 | 1.01 - 1.17 | 1.12 | 1.01 - 1.18 | 23,571 |  |
|  | dosage | 1.07 | 0.99 - 1.16 | 1.08 | 1.00 - 1.17 | 1.12 | 1.00 - 1.17 | 24,204 |  |
|  | quantity | 1.05 | 0.97 - 1.14 | 1.09 | 1.01 - 1.17 | 1.12 | 1.00 - 1.17 | 29,947 |  |
| Chew | count | 1.11 | 1.02 - 1.20 | 1.10 | 1.02 - 1.18 | 1.08 | 1.00 - 1.17 | 23,497 |  |
|  | value | 1.09 | 1.01 - 1.18 | 1.10 | 1.02 - 1.18 | 1.08 | 1.00 - 1.17 | 23,841 |  |
|  | dosage | 1.09 | 1.01 - 1.18 | 1.11 | 1.03 - 1.19 | 1.08 | 1.00 - 1.17 | 24,447 |  |
|  | quantity | 1.07 | 0.99 - 1.16 | 1.09 | 1.01 - 1.18 | 1.08 | 0.99 - 1.17 | 30,065 |  |
| Durán | count | 1.12 | 1.03 - 1.22 | 1.13 | 1.04 - 1.23 | 1.08 | 1.03 - 1.22 | 23,559 |  |
|  | value | 1.12 | 1.04 - 1.22 | 1.14 | 1.05 - 1.23 | 1.09 | 1.03 - 1.22 | 23,768 |  |
|  | dosage | 1.12 | 1.03 - 1.21 | 1.13 | 1.05 - 1.22 | 1.09 | 1.03 - 1.22 | 24,319 |  |
|  | quantity | 1.10 | 1.01 - 1.20 | 1.13 | 1.04 - 1.22 | 1.09 | 1.02 - 1.21 | 29,896 |  |
| Ehrt | count | 1.10 | 1.02 - 1.18 | 1.10 | 1.02 - 1.18 | 1.04 | 1.02 - 1.18 | 23,412 |  |
|  | value | 1.08 | 1.00 - 1.17 | 1.10 | 1.02 - 1.18 | 1.04 | 1.02 - 1.18 | 23,485 |  |
|  | dosage | 1.08 | 1.00 - 1.16 | 1.10 | 1.02 - 1.18 | 1.04 | 1.02 - 1.18 | 24,091 |  |
|  | quantity | 1.07 | 0.99 - 1.16 | 1.09 | 1.02 - 1.18 | 1.04 | 1.01 - 1.18 | 29,818 |  |
| Han | count | 1.04 | 0.96 - 1.13 | 1.05 | 0.97 - 1.14 | 1.09 | 0.97 - 1.13 | 23,577 |  |
|  | value | 1.05 | 0.97 - 1.14 | 1.06 | 0.98 - 1.14 | 1.09 | 0.97 - 1.14 | 23,730 |  |
|  | dosage | 1.04 | 0.96 - 1.13 | 1.04 | 0.97 - 1.13 | 1.10 | 0.97 - 1.13 | 24,270 |  |
|  | quantity | 1.05 | 0.97 - 1.15 | 1.05 | 0.97 - 1.14 | 1.09 | 0.97 - 1.14 | 29,926 |  |
| Kiesel | count | 1.10 | 1.02 - 1.20 | 1.11 | 1.02 - 1.20 | 1.05 | 1.01 - 1.20 | 23,561 |  |
|  | value | 1.11 | 1.03 - 1.21 | 1.12 | 1.03 - 1.21 | 1.06 | 1.02 - 1.21 | 23,750 |  |
|  | dosage | 1.11 | 1.02 - 1.21 | 1.10 | 1.02 - 1.20 | 1.06 | 1.01 - 1.20 | 29,894 |  |
|  | quantity | 1.11 | 1.02 - 1.21 | 1.10 | 1.02 - 1.20 | 1.05 | 1.01 - 1.20 | 29,894 |  |
| Rudolph | count | 1.08 | 1.00 - 1.16 | 1.07 | 1.00 - 1.15 | 1.10 | 1.00 - 1.16 | 23,487 |  |
|  | value | 1.07 | 0.99 - 1.16 | 1.07 | 1.00 - 1.16 | 1.10 | 1.00 - 1.16 | 23,529 |  |
|  | dosage | 1.08 | 0.99 - 1.17 | 1.07 | 0.99 - 1.15 | 1.10 | 1.00 - 1.17 | 24,060 |  |
|  | quantity | 1.05 | 0.97 - 1.15 | 1.07 | 0.99 - 1.16 | 1.11 | 0.99 - 1.16 | 29,881 |  |
| Sittironnarit | count | 1.05 | 0.97 - 1.15 | 1.07 | 0.99 - 1.16 | 1.06 | 0.98 - 1.15 | 23,534 |  |
|  | value | 1.07 | 0.99 - 1.17 | 1.07 | 0.99 - 1.16 | 1.06 | 0.99 - 1.16 | 23,678 |  |
|  | dosage | 1.06 | 0.97 - 1.15 | 1.06 | 0.98 - 1.15 | 1.06 | 0.98 - 1.16 | 24,293 |  |
|  | quantity | 1.06 | 0.98 - 1.16 | 1.07 | 0.99 - 1.16 | 1.06 | 0.98 - 1.16 | 30,097 |  |
| Polypharmacy | count | 1.06 | 0.94 - 1.12 | 1.06 | 0.97 - 1.16 | 1.05 | 0.95 - 1.15 | 24,103 |  |
| Polypharmacy plus | count | 1.02 | 0.98 - 1.15 | 1.02 | 0.93 - 1.13 | 1.02 | 0.92 - 1.12 | 24,103 |  |

**Supplementary Table 8** (see also next page): HRs for scaled (mean=0; standard deviation=1) numerical variables in the Cox proportional risks model predicting the risk of dementia by value-based scales. Each row depicts the effect on dementia risk according to a different predictor. The estimates for the covariates in the different models (that used different anticholinergic scales) did not differ substantially from one another and are thus not depicted for each model separately. Instead, they are depicted as ranges: within the parentheses a range is given that was observed across all models. To fit model prerequisites, polypharmacy is transformed by taking the square root before scaling.

| **Variable** | **Level** | **HR** | **95% CI** | **99.9% CI** |
| --- | --- | --- | --- | --- |
| AChB |  | [1.02 , 1.12] | [1.00, 1.10] -  [1.05, 1.15] | [0.94, 1.04] -  [1.12, 1.21] |
| Polypharmacy |  | [1.01, 1.04] | [1.00, 1.03] -  [1.03, 1.06] | [0.96, 1.00] -  [1.04, 1.09] |
| Sex | Male | [1.44, 1.47] | [1.37, 1.39] -  [1.52, 1.55] | [1.21, 1.23] -  [1.72, 1.75] |
| Data provider | Scotland | [0.22, 0.24] | [0.18, 0.20] -  [0.28, 0.30] | [0.11, 0.12] -  [0.45, 0.48] |
|  | England (TPP) | [1.40, 1.45] | [1.26, 1.30] -  [1.56, 1.61] | [0.99, 1.03] -  [1.98, 2.04] |
|  | Wales | [0.52, 0.53] | [0.44, 0.45] -  [0.61, 0.63] | [0.31, 0.32] -  [0.87, 0.89] |
| Age |  | [1.22, 1.22] | [1.21, 1.21] -  [1.22, 1.23] | [1.19, 1.20] -  [1.24, 1.25] |
| Year |  | [0.98, 0.98] | [0.97, 0.97] -  [0.99, 0.99] | [0.94, 0.95] -  [1.01, 1.02] |
| Education | Graduate degree | [0.81, 0.82] | [0.76, 0.77] -  [0.87, 0.88] | [0.66, 0.67] -  [1.00, 1.01] |
| Deprivation |  | [1.03, 1.03] | [1.02, 1.02] -  [1.04, 1.04] | [1.00, 1.00] -  [1.06, 1.06] |
| BMI | 18.5-25 | [0.64, 0.71] | [0.47, 0.52] -  [0.87, 0.98] | [0.24, 0.25] -  [1.73, 2.01] |
|  | 25-30 | [0.54, 0.59] | [0.40, 0.43] -  [0.73, 0.82] | [0.20, 0.21] -  [1.44, 1.67] |
|  | 30-35 | [0.51, 0.57] | [0.37, 0.41] -  [0.69, 0.78] | [0.19, 0.20] -  [1.38, 1.61] |
|  | 35-40 | [0.55, 0.61] | [0.40, 0.43] -  [0.76, 0.85] | [0.19, 0.20] -  [1.56, 1.81] |
|  | >40 | [0.49, 0.56] | [0.34, 0.38] -  [0.71, 0.83] | [0.14, 0.16] -  [1.66, 1.97] |
| Smoking | Previous smoker | [1.13, 1.14] | [1.07, 1.08] -  [1.19, 1.20] | [0.94, 0.95] -  [1.34, 1.36] |
|  | Current smoker | [1.25, 1.28] | [1.14, 1.17] -  [1.37, 1.41] | [0.93, 0.95] -  [1.69, 1.73] |
| Alcohol consumption | Three or four times a week | [0.83, 0.85] | [0.77, 0.79] -  [0.89, 0.92] | [0.65, 0.67] -  [1.06, 1.09] |
|  | Once or twice a week | [0.90, 0.91] | [0.83, 0.84] -  [0.96, 0.98] | [0.71, 0.72] -  [1.14, 1.15] |
|  | Once to three times a month | [0.89, 0.91] | [0.81, 0.82] -  [0.99, 1.00] | [0.65, 0.66] -  [1.23, 1.25] |
|  | Only special occasions | [1.12, 1.15] | [1.03, 1.05] -  [1.22, 1.26] | [0.84, 0.87] -  [1.49, 1.53] |
|  | Never | [1.33, 1.38] | [1.21, 1.26] -  [1.46, 1.51] | [0.99, 1.02] -  [1.80, 1.86] |
| Physical activity | Moderate | [0.75, 0.76] | [0.71, 0.72] -  [0.79, 0.81] | [0.63, 0.64] -  [0.89, 0.91] |
|  | Strenuous | [0.64, 0.66] | [0.56, 0.58] -  [0.73, 0.75] | [0.42, 0.44] -  [0.97, 0.99] |
| Number of prior comorbidities |  | [1.0, 1.0] | [1.00, 1.00] -  [1.00, 1.00] | [1.00, 1.00] -  [1.00, 1.00] |
| Prior depression |  | [1.15, 1.29] | [1.05, 1.18] -  [1.25, 1.40] | [0.87, 0.98] -  [1.52, 1.69] |
| Prior stroke |  | [1.46, 1.56] | [1.21, 1.30] -  [1.75, 1.86] | [0.80, 0.88] -  [2.64, 2.78] |
| Prior diabetes |  | [1.69, 1.86] | [1.49, 1.65] -  [1.91, 2.11] | [1.13, 1.26] -  [2.51, 2.78] |
| Prior hypercholesterolemia |  | [1.03, 1.08] | [0.91, 0.95] -  [1.17, 1.22] | [0.69, 0.72] -  [1.54, 1.61] |
| Prior hypertension |  | [1.20, 1.25] | [1.11, 1.16] -  [1.30, 1.35] | [0.94, 0.98] -  [1.53, 1.60] |
| APOE carrier | ε2 | [0.82 ,0.84] | [0.74, 0.77] -  [0.90, 0.93] | [0.60, 0.62] -  [1.13, 1.16] |
|  | ε4 | [2.72, 2.76] | [2.58, 2.62] -  [2.87, 2.90] | [2.30, 2.33] -  [3.22, 3.26] |

| **Variable** | **Level** | **HR** | **99% CI** |
| --- | --- | --- | --- |
| AChB |  | 1.12 | 1.04 - 1.22 |
| Sex | Male | 1.46 | 1.22 - 1.75 |
| Year 0 |  | 0.98 | 0.95 - 1.02 |
| Age 0 |  | 1.22 | 1.19 - 1.24 |
| Education | Graduate degree | 0.82 | 0.67 - 1.02 |
| Deprivation |  | 1.03 | 1.00 - 1.06 |
| Alcohol consumption | Three or four times a week Once or twice a week Once to three times a month Only special occasions Never | 0.83 0.90 0.90 1.13 1.34 | 0.65 - 1.07 0.71 - 1.14 0.65 - 1.25 0.85 - 1.51 0.99 - 1.83 |
| Smoking | Current smoker  Previous smoker | 1.13 1.26 | 0.94 - 1.35 0.93 - 1.72 |
| Physical activity | Strenuous  Moderate | 0.76 0.66 | 0.64 - 0.92 0.43 - 1.00 |
| BMI | <18.5 25-30 30-35 35-40 >40 | 0.65 0.54 0.51 0.55 0.50 | 0.23 - 1.78 0.20 - 1.48 0.18 - 1.42 0.19 - 1.61 0.14 - 1.74 |
| Data provider | England TPP Scotland Wales | 1.43 0.24 0.53 | 1.01 - 2.03 0.12 - 0.48 0.31 - 0.90 |
| Prior depression |  | 1.18 | 0.89 - 1.57 |
| Prior stroke |  | 1.54 | 0.85 - 2.79 |
| Prior diabetes |  | 1.84 | 1.23 - 2.75 |
| Prior hypercholesterolemia |  | 1.06 | 0.70 - 1.59 |
| Prior hypertension |  | 1.25 | 0.97 - 1.61 |
| Number of prior comorbidities |  | 1 | 1.00 - 1.00 |
| Non-anticholinergic drug count |  | 1.02 | 0.97 - 1.07 |
| *APOE* carrier | ε2  ε4 | 0.83 2.73 | 0.60 - 1.15 2.30 - 3.25 |

**Supplementary Table 9**: HRs for scaled (mean=0; standard deviation=1) numerical variables in the Cox proportional risks model predicting the risk of dementia. Anticholinergic burden was determined using the value-based scale by Durán et al. (2013)^5^.

**Supplementary Table 10**: HRs for scaled numerical variables in the Cox proportional risks model predicting the risk of dementia. Each row depicts the effect of anticholinergic burden due to a drug prescribed for a different anatomical group.

| **Anatomical group** | **HR** | **95% CI** | **n missing** |
| --- | --- | --- | --- |
| nervous | 1.12 | 1.07 - 1.18 | 19,700 |
| cardiovascular | 1.05 | 1.02 - 1.09 | 19,700 |
| gastrointestinal | 1.05 | 1.00 - 1.09 | 19,700 |
| blood | 1.02 | 1.00 - 1.05 | 19,700 |
| hormonal | 1.02 | 0.97 - 1.07 | 19,700 |
| respiratory | 1.01 | 0.96 - 1.06 | 19,700 |
| antiinfective | 1.01 | 0.97 - 1.06 | 19,700 |
| urinary | 0.99 | 0.95 - 1.04 | 19,700 |
| immuno-modulating | 0.99 | 0.94 - 1.04 | 19,700 |
| musculo-skeletal | 0.99 | 0.94 - 1.03 | 19,700 |

| **Pharmacological group** | **HR** | **95% CI** | **n missing** |
| --- | --- | --- | --- |
| antidepressant | 1.11 | 1.07 - 1.17 | 20,047 |
| antiepileptic | 1.07 | 1.04 - 1.11 | 20,047 |
| high ceiling diuretic | 1.06 | 1.02 - 1.10 | 20,047 |
| acid reflux | 1.04 | 0.99 - 1.08 | 20,047 |
| propulsive | 1.03 | 0.99 - 1.08 | 20,047 |
| antipsychotic | 1.02 | 0.98 - 1.07 | 20,047 |
| corticosteroid | 1.02 | 0.98 - 1.07 | 20,047 |
| decongestant | 1.02 | 0.98 - 1.06 | 20,047 |
| cardiac Ca-blocker | 1.02 | 0.98 - 1.05 | 20,047 |
| antithrombotic | 1.02 | 0.99 - 1.04 | 20,047 |
| antihistamine | 1.01 | 0.96 - 1.06 | 20,047 |
| antipropulsive | 1.01 | 0.97 - 1.05 | 20,047 |
| penicillin | 1.01 | 0.96 - 1.06 | 20,047 |
| anxiolytic | 1.01 | 0.96 - 1.05 | 20,047 |
| glucose-lowering | 1.01 | 0.96 - 1.06 | 20,047 |
| vasodilator | 1.00 | 0.96 - 1.04 | 20,047 |
| cardiac glycoside | 0.99 | 0.95 - 1.04 | 20,047 |
| opioid | 0.99 | 0.94 - 1.04 | 20,047 |
| immunosuppressant | 0.99 | 0.93 - 1.04 | 20,047 |
| urological | 0.99 | 0.94 - 1.04 | 20,047 |
| sedative | 0.98 | 0.93 - 1.03 | 20,047 |
| antimigraine | 0.98 | 0.92 - 1.03 | 20,047 |

**Supplementary Table 11**: HRs for scaled numerical variables in the Cox proportional risks model predicting the risk of dementia. Each row depicts the effect of anticholinergic burden due to a drug prescribed for a different pharmacological group.

**Supplementary Table 12**: HRs for scaled numerical variables in the Cox proportional risks model predicting the risk of dementia. Each row depicts the effect of anticholinergic burden due to a drug prescribed for a different category of anticholinergic potency. The last column indicates the number of participants that were issued at least one prescription from each potency group.

| **Potency score** | **HR** | **95% CI** | **n missing** | **n > 0** |
| --- | --- | --- | --- | --- |
| 2 | 1.03 | 0.98 - 1.07 | 20,693 | 7,745 |
| 1 | 1.10 | 1.05 - 1.15 | 20,693 | 36,500 |
| 0.5 | 1.03 | 0.99 - 1.08 | 20,693 | 35,120 |
| 0 | 1.03 | 0.98 - 1.09 | 20,693 | 117,583 |

**References**

1. Carnahan RM, Lund BC, Perry PJ, Pollock BG, Gulp KR. The anticholinergic drug scale as a measure of drug-related anticholinergic burden: Associations with serum anticholinergic activity. *J Clin Pharmacol*. 2006;46(12):1481-1486. doi:10.1177/0091270006292126

2. Boustani M, Campbell N, Munger S, Maidment I, Fox C. Impact of anticholinergics on the aging brain: A review and practical application. *Aging health*. 2008;4(3):311-320. doi:10.2217/1745509X.4.3.311

3. Rudolph JL, Salow MJ, Angelini MC, McGlinchey RE. The anticholinergic risk scale and anticholinergic adverse effects in older persons. *Arch Intern Med*. 2008;168(5):508-513. doi:10.1001/archinternmed.2007.106

4. Sumukadas D, McMurdo MET, Mangoni AA, Guthrie B. Temporal trends in anticholinergic medication prescription in older people: Repeated crosssectional analysis of population prescribing data. *Age Ageing*. 2013;0:1-7. doi:10.1093/ageing/aft199

5. Durán CE, Azermai M, Stichele RHV. Systematic review of anticholinergic risk scales in older adults. *Eur J Clin Pharmacol*. 2013;69(7):1485-1496. doi:10.1007/s00228-013-1499-3

6. Summers WK. A clinical method of estimating risk of drug induced delirium. *Life Sci*. 1978;22:1511-1516.

7. Han L, McCusker J, Cole M, Abrahamowicz M, Primeau F, Élie M. Use of medications with anticholinergic effect predicts clinical severity of delirium symptoms in older medical inpatients. *Arch Intern Med*. 2001;161(8):1099-1105. doi:10.1001/archinte.161.8.1099

8. Han L, Agostini J V., Allore HG. Cumulative anticholinergic exposure is associated with poor memory and executive function in older men. *J Am Geriatr Soc*. 2008;56(12):2203-2210. doi:10.1111/j.1532-5415.2008.02009.x

9. Aizenberg D, Sigler M, Weizman A, Barak Y. Anticholinergic burden and the risk of falls among elderly psychiatric inpatients: A 4-year case-control study. *Int Psychogeriatrics*. 2002;14(3):307-310. doi:10.1017/S1041610202008505

10. Minzenberg MJ, Poole JH, Benton C, Vinogradov S. Assocation of Anticholinergic Load with Impairment of Complex Attention and Memory in Schizophrenia. *Am J Psychiatry*. 2004;161(1):116-124. doi:10.1176/appi.ajp.161.1.116

11. Ancelin ML, Artero S, Portet F, Dupuy AM, Touchon J, Ritchie K. Non-degenerative mild cognitive impairment in elderly people and use of anticholinergic drugs: Longitudinal cohort study. *Br Med J*. 2006;332(7539):455-458. doi:10.1136/bmj.38740.439664.DE

12. Hilmer SN, Mager DE, Simonsick EM, et al. A drug burden index to define the functional burden of medications in older people. *Arch Intern Med*. 2007;167(8):781-787. doi:10.1001/archinte.167.8.781

13. Chew ML, Mulsant BH, Pollock BG, et al. Anticholinergic activity of 107 medications commonly used by older adults. *J Am Geriatr Soc*. 2008;56(7):1333-1341. doi:10.1111/j.1532-5415.2008.01737.x

14. Cancelli I, Valentinis L, Merlino G, Valente M, Gigli GL. Drugs with anticholinergic properties as a risk factor for psychosis in patients affected by Alzheimer’s disease. *Clin Pharmacol Ther*. 2008;84(1):63-68. doi:10.1038/sj.clpt.6100435

15. Ehrt U, Broich K, Larsen JP, Ballard C, Aarsland D. Use of drugs with anticholinergic effect and impact on cognition in Parkinson’s disease: A cohort study. *J Neurol Neurosurg Psychiatry*. 2010;81(2):160-165. doi:10.1136/jnnp.2009.186239

16. Sittironnarit G, Ames D, Bush AI, et al. Effects of anticholinergic drugs on cognitive function in older Australians: Results from the AIBL study. *Dement Geriatr Cogn Disord*. 2011;31(3):173-178. doi:10.1159/000325171

17. Whalley LJ, Sharma S, Fox HC, et al. Anticholinergic drugs in late life: Adverse effects on cognition but not on progress to dementia. *J Alzheimer’s Dis*. 2012;30(2):253-261. doi:10.3233/JAD-2012-110935

18. Faure R, Dauphinot V, Krolak-Simon, P., et al. A standard international version of the drug burden index for cross-national comparison of the functional burden of medications in older people. *Journal of the American Geriatrics Society*. 2013;61(7):1227-1228. doi: 10.1111/jgs.12343

19. Klamer TT, Wauters M, Azermai M, et al. A Novel Scale Linking Potency and Dosage to Estimate Anticholinergic Exposure in Older Adults: the Muscarinic Acetylcholinergic Receptor ANTagonist Exposure Scale. *Basic Clin Pharmacol Toxicol*. 2017;120(6):582-590. doi:10.1111/bcpt.12699

20. Bishara D, Harwood D, Sauer J, Taylor DM. Anticholinergic effect on cognition (AEC) of drugs commonly used in older people. *Int J Geriatr Psychiatry*. 2017;32(6):650-656. doi:10.1002/gps.4507

21. Briet J, Javelot H, Heitzman E, et al. The anticholinergic impregnation scale: towards the elaboration of a scale adapted to prescriptions in French psychiatric settings. *Therapie*. 2017;72:427-437. doi:10.1016/j.therap.2016.12.010

22. Kiesel EK, Hopf YM, Drey M. An anticholinergic burden score for German prescribers: Score development. *BMC Geriatr*. 2018;18(1):1-11. doi:10.1186/s12877-018-0929-6
